# Genome-wide identification and gene expression profiling of ubiquitin ligases for endoplasmic reticulum protein degradation

**DOI:** 10.1038/srep30955

**Published:** 2016-08-03

**Authors:** Masayuki Kaneko, Ikuko Iwase, Yuki Yamasaki, Tomoko Takai, Yan Wu, Soshi Kanemoto, Koji Matsuhisa, Rie Asada, Yasunobu Okuma, Takeshi Watanabe, Kazunori Imaizumi, Yausyuki Nomura

**Affiliations:** 1 Department of Biochemistry, Institute of Biomedical and Health Sciences, Hiroshima University, Hiroshima 734-8553, Japan; 2 Department of Pharmacology, Graduate School of Pharmaceutical Sciences, Hokkaido University, Sapporo 060-0812, Japan; 3 Otsuka GEN Research Institute, Otsuka Pharmaceutical Co., Ltd., Tokushima 771-0192, Japan; 4 Department of Biochemistry, Graduate school of Biomedical and Health Sciences, Hiroshima University, Hiroshima 734-8553, Japan; 5 Department of Pharmacology, Faculty of Pharmaceutical Sciences, Chiba Institute of Science, Choshi, Chiba 288-0025, Japan; 6 Department of Pharmacology, Kurume University School of Medicine, Kurume, Fukuoka 830-0011, Japan

## Abstract

Endoplasmic reticulum (ER)-associated degradation (ERAD) is a mechanism by which unfolded proteins that accumulate in the ER are transported to the cytosol for ubiquitin–proteasome-mediated degradation. Ubiquitin ligases (E3s) are a group of enzymes responsible for substrate selectivity and ubiquitin chain formation. The purpose of this study was to identify novel E3s involved in ERAD. Thirty-seven candidate genes were selected by searches for proteins with RING-finger motifs and transmembrane regions, which are the major features of ERAD E3s. We performed gene expression profiling for the identified E3s in human and mouse tissues. Several genes were specifically or selectively expressed in both tissues; the expression of four genes (RNFT1, RNF185, CGRRF1 and RNF19B) was significantly upregulated by ER stress. To determine the involvement of the ER stress-responsive genes in ERAD, we investigated their ER localisation, *in vitro* autoubiquitination activity and ER stress resistance. All were partially localised to the ER, whereas CGRRF1 did not possess E3 activity. RNFT1 and RNF185, but not CGRRF1 and RNF19B, exhibited significant resistance to ER stressor in an E3 activity-dependent manner. Thus, these genes are possible candidates for ERAD E3s.

The ubiquitin–proteasome system (UPS) is an important mechanism for protein degradation in the cytosol and nucleus. The UPS is conserved from yeast to mammals and is involved in a wide variety of cellular events, such as cell proliferation, transcriptional regulation, apoptosis, immunity and development^1^,^2^. Dysfunction in the system has been linked to the pathogenesis of various human diseases^3^,^4^. Ubiquitination is mediated by a set of enzymes in an ATP-dependent manner. Ubiquitin activating enzyme (E1) forms a thioester bond with ubiquitin using ATP to transfer the activated ubiquitin to ubiquitin conjugating enzyme (E2). The E2-conjugated ubiquitin is then transferred to a lysine residue on the substrate protein, forming an isopeptide bond. This reaction is mediated by ubiquitin ligase (E3) and repeated to form polyubiquitin chains. Polyubiquitinated proteins are eventually targeted to the 26S proteasome for degradation^1^,^5^. E3 ligase is the most critical enzyme in the UPS, since E3 provides substrate specificity and controls degradation speed as the rate-limiting enzyme.

Hundreds of E3 ligases are estimated to exist in the human genome, to ubiquitinate a subset of substrate proteins^6^. E3 ligases are classified into three major groups on the basis of their specific structural motifs: RING-finger, HECT or U-box^5^,^6^. Among them, the RING-finger type is the major group, estimated to include more than 600 members^5^. The RING structure coordinates two zinc ions (Zn^2+^) with eight residues, generally consisting of Cys and His. Canonical RINGs contain either one or two His in the coordinating residues and are classified into two major subtypes: C3H2C3 or C3HC4 (represented by the sequence)^5^,^7^.

Endoplasmic reticulum (ER)-associated degradation (ERAD) is a protective mechanism against ER stress. In ERAD, unfolded proteins that have accumulated in the ER are selectively transported to the cytosol for degradation by the UPS^8^. In yeast, only three E3 ligases, Hrd1, Doa10 and Asi, are involved in ERAD^9^,^10^,^11^,^12^. We identified the human ERAD E3 ligase, HRD1 (also called SYVN1), based on the protein sequence of yeast homolog, Hrd1^13^. HRD1 possesses a RING-finger domain and 6 transmembrane domains including a signal peptide. Human HRD1 is upregulated in response to ER stress and localises to the ER, consistent with its yeast homolog^13^,^14^.

There has been speculation that many more ERAD E3s exist in mammals because hundreds have been predicted to be present in their genomes^5^. However, it remains to be determined whether many mammalian E3s are involved in ERAD. In the present study, using *in silico* approaches, we identified 37 E3 ligases containing RING-finger and transmembrane domains, which are potentially involved in ERAD. We experimentally investigated the tissue distribution of the identified E3 ligases and eventually identified two candidates for E3 ligases involved in ERAD by demonstrating their responsiveness and resistance to ER stress.

## Results

### Identification of E3 ligases containing RING-finger and transmembrane domains

To identify human E3 ligases that are potentially involved in ERAD, we searched human protein databases (RefSeq and Ensembl), using the following criteria: (1) having a RING-finger motif (C3H2C3 or C3HC4) and (2) having transmembrane domain(s). We found a total of 37 putative RING-type E3 genes with transmembrane domain(s) matching these criteria, 19 containing C3H2C3 motifs (Table 1) and 18 with C3HC4 motifs (Table 2). The number of transmembrane spans, which is predicted by TMHMM, ranges from 1 to 12 times (Tables 1 and 2; Supplementary Fig. S1). The search results demonstrated that a known ERAD E3 ligase, HRD1/SYVN1, was selected using this search criteria (Table 1).

Lists were aligned based on relatedness via multiple sequence alignment using CLUSTALW (Supplementary Fig. S2). Furthermore, to clarify structural and functional homology between identified genes, we constructed additional phylogenetic trees using the TreeFam database (Supplementary Fig. S3). The same gene families are grouped into the same symbol based on the gene tree (Tables 1 and 2).

### Tissue distribution of transmembrane E3 ligases

**Figure 1:**
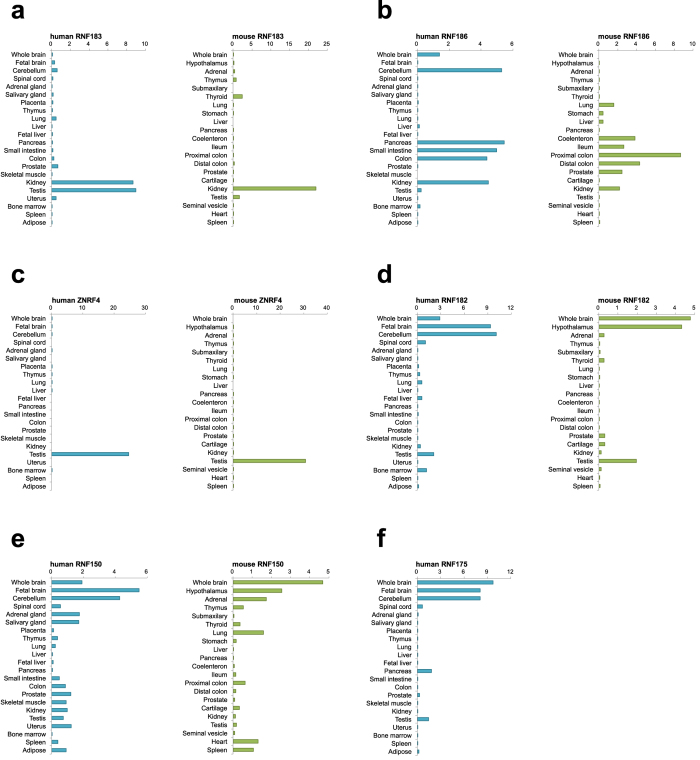
Tissue-specific distribution of transmembrane E3 ligases. Total RNAs from 23 human or 22 mouse tissues were reverse-transcribed and measured by TaqMan-based real-time PCR assay using the delta–delta Ct method. Data are normalised to the amount of 18S ribosomal RNA; results are expressed as the fold increase compared with cDNA pools from each tissue. (**a**) RNF183, (**b**) RNF186, (**c**) ZNRF4, (**d**) RNF182, (**e**) RNF150, (**f**) RNF175.

To characterise the physiological roles of identified E3s, we investigated the tissue distribution of the products of the 37 genes using total RNA from 22 normal human and 23 normal mouse tissues. These results indicate that a majority of the genes were ubiquitously expressed (Supplementary Figs S4 and S5; Table S1). On the other hand, several genes were specifically (a single tissue) or selectively (limited tissues) expressed in both human and mouse tissues; RNF183 (kidney; Fig. 1a), RNF186 (lower gastrointestinal tract; Fig. 1b), ZNRF4 (testis; Fig. 1c), RNF182 (neural tissues; Fig. 1d) and RNF150 (neural tissues; Fig. 1d). RNF175 is a human-specific gene and highly expressed in neural tissues (Fig. 1e).

### ER stress responsiveness of the transmembrane E3 ligases

As some ERAD components, including HRD1/SYVN1, SEL1L and Derlin-1, are upregulated in response to ER stress^13^,^14^, we hypothesised that transmembrane E3 ligases expressed during ER stress are potentially involved in ERAD. The ER stress transducer ATF6 is processed in response to ER stress and subsequently translocates from the ER to the nucleus as a transcription factor. The processed ATF6 binds to the ER stress response element (ERSE)-I and -II, CCAAT-N_9_-CCACG/A and ATTGG-N-CCACG, respectively^15^,^16^,^17^. Another UPR transducer IRE1 activates by trans-autophosphorylation during ER stress and splices XBP1 mRNA depending on its ribonuclease activity. The spliced XBP1 encodes another open reading frame and binds to the mammalian unfolded protein response element (UPRE; TGACGTGG/A) as a transcription factor^17^,^18^. First, in an attempt to computationally identify ER stress-responsive genes, we searched for ER stress-responsive motifs, ERSE (CCAAT-N_9_-CCACG/A), ERSE-II (ATTGG-N-CCACG) or UPRE (TGACGTGG/A), in the 5′ upstream region of the E3 genes identified (Tables 1 and 2). These consensus sequences were found in the following 8 genes: RNF130 (UPRE, -315), RNF149 (ERSE-I, -2586), RNF145 (ERSE-II, -1258), HRD1 (ERSE-I, -506; UPRE, -2303), RNF121 (UPRE, -2372), RNF19A (UPRE, -428), RNF170 (UPRE, -627) and RNF180 (ERSE-II, -4801).

**Figure 2:**
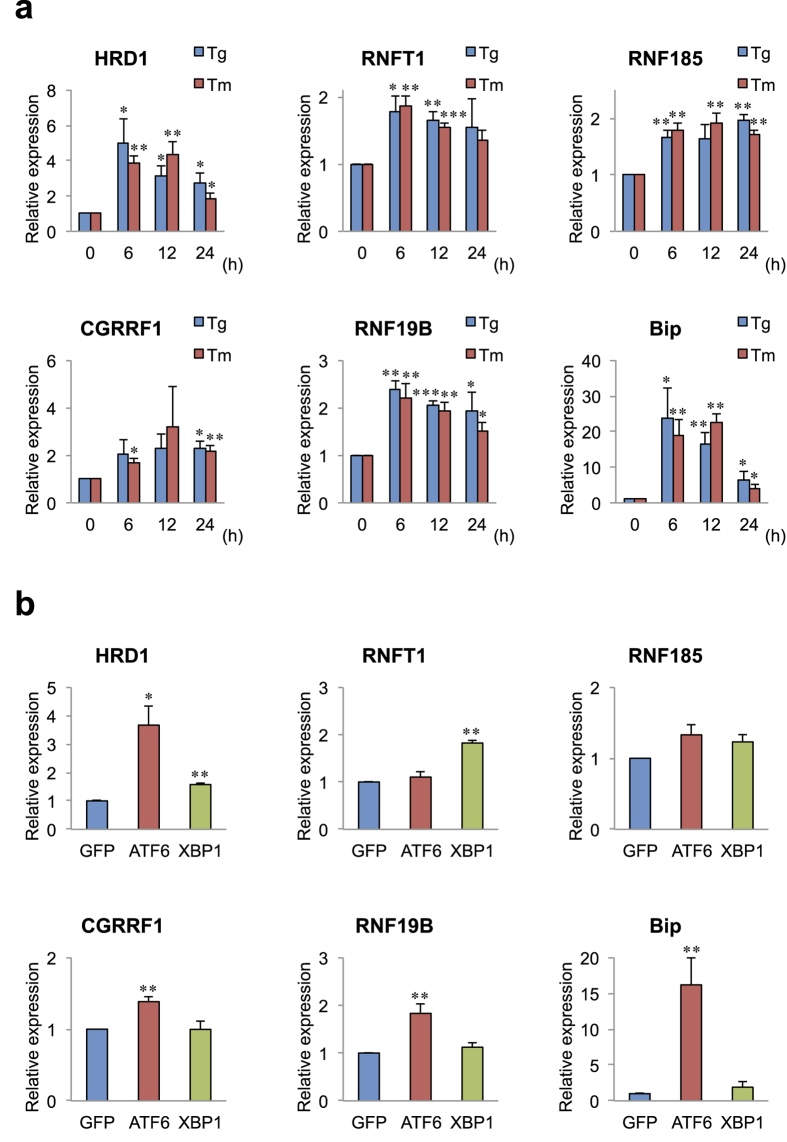
ER stress response of transmembrane E3 ligases (**a**) Expression of E3 ligases induced by ER stressor. HeLa cells were treated with thapsigargin (Tg; 1 μM) and tunicamycin (Tm; 3 μg/ml) for the time periods as indicated. Total RNAs were reverse-transcribed and measured by TaqMan-based real-time PCR assay using the delta–delta Ct method. Data are normalized to the amount of GAPDH; results are expressed as the fold increase compared with control (mean ± SD; n = 4). (**b**) Expression of E3 ligases induced by the overexpression of ATF6 and XBP1. pcDNA3–ATF6α (1–373 amino acid residues),–XBP1 (spliced form), or–GFP (as a control) were transfected into HeLa cells. The cells were incubated for 36 h. Data are normalized to the amount of GAPDH; results are expressed as the fold increase compared with GFP (mean ± SD; n = 3). Statistical analysis was performed with ANOVA followed by Bonferroni correction (vs. control and GFP; *p < 0.05, **p < 0.01, ***p < 0.001).

To confirm predictions of ER stress responsiveness and to identify ERAD E3s from among the 37 genes, we experimentally tested whether the expression of these genes is induced by ER stressors, thapsigargin (Tg; ER Ca^2+^-ATPase inhibitor) and tunicamycin (Tm; N-glycosylation inhibitor), in HeLa cells, a well-characterised cell line where the three major UPR pathways are active and highly responsiveness to ER stress. The expression of four genes RNFT1, RNF185, CGRRF1 and RNF19B was significantly elevated by both Tg and Tm, although the induction levels of these genes (approximately two-fold) were lower than those of HRD1 (approximately four-fold) or Bip (an ER chaperone; approximately 20-fold), used as positive controls (Fig. 2a; Supplementary Fig. S6a,b). The expression of RNFT1 and RNF19B was elevated with a peak at 6 h as HRD1 and Bip. On the other hand, RNF185 and CGRRF1 were probable upregulated via indirect UPR pathways because their peaks occurred at 24 or 48 h, respectively.

We previously demonstrated that HRD1 expression is induced by ATF6 and XBP1 overexpression^14^. To determine which transcription factors are required for the expression of these ER stress-induced E3 genes, we examined the effect of ATF6 and XBP1 overexpression on the ER stress-responsive E3 genes. ATF6 (N-terminal domain) significantly upregulated CGRRF1 and RNF19B expression, although the induction levels of these genes (less than two-fold) were lower than those of HRD1 (approximately five-fold) (Fig. 2b). On the other hand, XBP1 (spliced form) significantly upregulated RNFT1 expression to the same extent (approximately two-fold) (Fig. 2b). Consistent with previous results^14^, HRD1 expression was induced by both ATF6 and XBP1 (Fig. 2b).

### Involvement of transmembrane E3 ligases in ERAD

**Figure 3:**
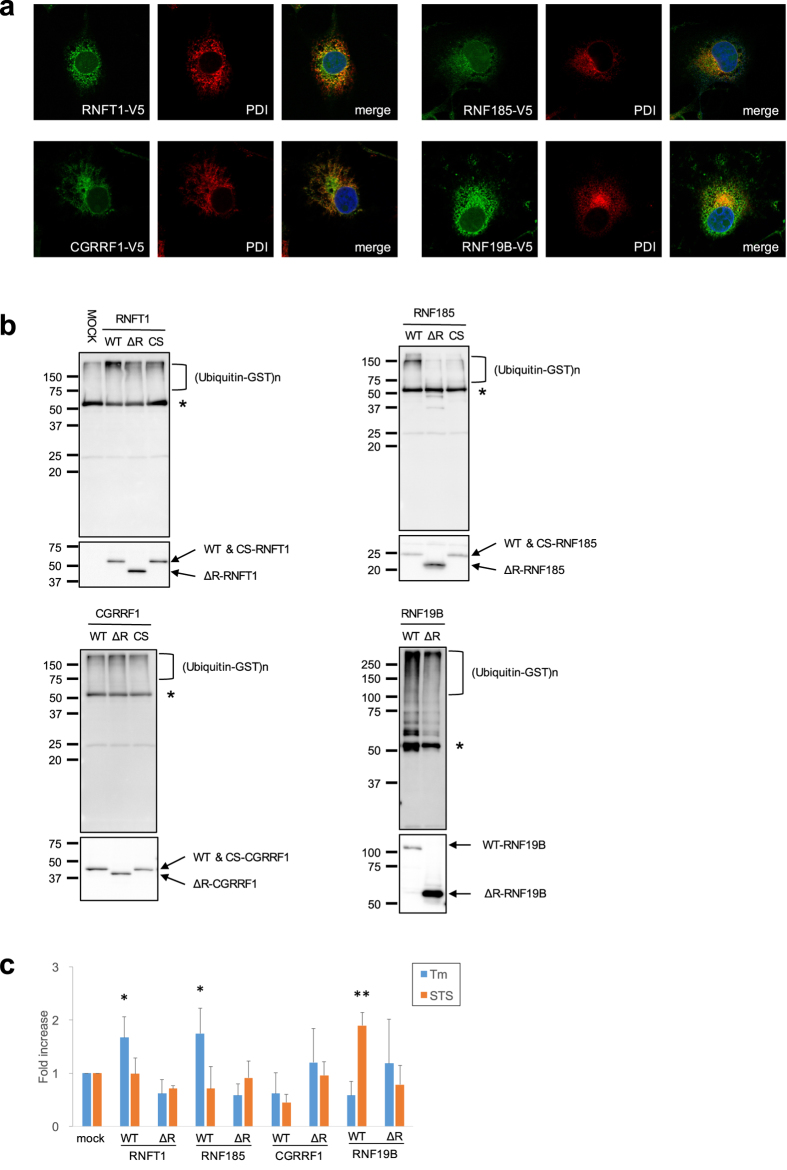
Characterization of candidates for ERAD E3 ligase (**a**) Subcellular localisation of E3 ligases. COS-1 cells stably expressing E3-V5 were subjected to immunofluorescence staining with anti-V5 and -PDI antibodies. (**b**) *In vitro* autoubiquitination assay. The E3 proteins produced by a transcription/translation system were immunoprecipitated with anti-V5 antibody, and then mixed in the reaction buffer with E1 (GST-tagged), E2 (GST-UbcH5c) and HA-ubiquitin. The reaction mixture was again immunoprecipitated with anti-V5 antibody and analysed via Western blotting using anti-ubiquitin or V5-antibodies. CS mutants defective in E3 activity were constructed by replacement of conserved coordinating Cys with Ser residues in the RING. Asterisk indicates the heavy chain of immunoglobulin. (**c**) E3 ligases protect against ER stress-induced cell death. Neuro-2a (N2a) cells stably expressing WT or ΔRING (ΔR) mutant of E3 ligases were transiently treated with Tm (1 μg/ml) or staurosporine (STS; 0.1 μM) and incubated for 48 h. The cells were stained with crystal violet. The eluted dye at an optical density of 590 nm was measured. Cell viability was calculated as follows: OD for assay/OD for vehicle control (0.1% dimethyl sulfoxide) well. The results are expressed as the fold increase compared with mock cells, in terms of means ± SD (three independent experiments in duplicate). Statistical analysis was performed using ANOVA followed by Bonferroni correction (vs. controls; *p < 0.05, **p < 0.01)

To assess whether the ER stress-responsive E3s are involved in ERAD, we determined the subcellular localisation of the E3s. The stable overexpression of V5-tagged E3s in COS-1 cells revealed that RNFT1, RNF185, CGRRF1 and RNF19B partially colocalised with endogenous PDI, an ER-resident chaperone, indicating their localisation to the ER (Fig. 3a).

Next, to demonstrate their E3 activity, we performed *in vitro* autoubiquitination assays using wild-type (WT) and mutant E3s in the presence of E1, E2 and ubiquitin. WT-E3s, RNFT1, RNF185 and RNF19B exhibited smears of high-molecular-weight bands detected by anti-multiubiquitin antibody, indicating ubiquitinated proteins. Their deleted RING (ΔR) and point mutations (CS) of RING-finger domain showed decreased levels of those smears (Fig. 3b). However, the smears of WT-CGRRF1 remained at basal levels compared with those in its mutants, indicating that CGRRF1 does not have E3 activity, at least in an *in vitro* system using the UbcH5c E2 enzyme (Fig. 3b).

The overexpression of HRD1 protects cells form ER stress-induced apoptosis, because HRD1 can promote the degradation of unfolded proteins that accumulate in the ER through the ERAD pathway^13^. To determine the involvement of transmembrane E3 ligases in ERAD, neuroblastoma Neuro-2a (N2a) cells, which are vulnerable to ER stress and can be easily induced to undergo apoptosis, overexpressing WT or ΔR mutant E3s were exposed to the ER stressor Tm or the non-ER stressor staurosporine (STS) (Fig. 3c). We found that the overexpression of WT-RNFT1 and RNF185 exhibited significant resistance to ER stress compared with mock cells transfected with empty vectors, while ΔR mutants failed to protect cells from ER stress. Furthermore, WT-RNFT1 and RNF185 did not exhibit resistance to STS-induced cell death. In contrast, WT-RNF19B did not exhibit resistance to ER stress, although it protected against STS-induced cell death. Consistent with the lack of E3 activity, WT-CGRRF1 had no resistance to ER stress. These results suggest that RNFT1 and RNF185 are potentially involved in ERAD in a similar manner as HRD1.

## Discussion

We identified 37 human genes encoding RING-finger and transmembrane domains over ten years ago. Recently, we found additional candidate genes using phylogenetic tree analysis (Supplementary Fig. S3): RNF148 (RNF130 family), RNF144A and RNF144B (RNF19A family), RNF223 (RNF183 family) and RNFT2 (RNFT1 family). On the other hand, Nakamura *et al.*^19^ and Neutzner *et al.*^20^ reported the 37 E3s identified by us as well as additional RING-finger E3s containing transmembrane domains, which were identified as a membrane-associated RING-CH (MARCH) family consisting of seven genes. Our search failed to identify these genes because the MARCH family has another minor RING-finger, C4HC3/RING variant (RINGv)^21^. Furthermore, RING-C2 E3, a non-canonical RING which has Cys at all of its coordinating residues, can also be involved in ERAD. RING-C2 E3 TMEM129, which is associated with the ERAD component Derlin-1, promotes a virus-induced degradation of MHC-I through the ERAD pathway^22^. Therefore, further various types of RING E3s most likely exist in the mammalian ERAD system.

ERAD has generally focused on the molecular mechanism of yeast. In humans, disruption of the ER quality control system, called ER stress, causes various diseases, such as neurodegenerative disease, metabolic disorders, inflammatory diseases and cancer^3^,^23^. Furthermore, ER stress has gained further interest because of its role in cellular differentiation and tissue development^24^,^25^. As for the reason why so many E3 genes would exist in mammals compared with the only three yeast ubiquitin ligases involved in ERAD (Hrd1, Doa10, and Asi1)^10^, it is assumed that they have tissue-specific and/or developmental stage-specific roles. Thus, we investigated the tissue distribution of the human E3 genes identified, and indeed, found several tissue-specific/selective E3s. This is the first study reporting the tissue-specific expression of transmembrane E3s (RNF183, RNF186, ZNRF4, RNF150 and RNF175), whose functions have not yet been known. RNF182 has previously been reported to be preferentially expressed in neural tissues, consistent with our result^26^. Although RNF182 expression is elevated in post-mortem Alzheimer’s disease brain^26^, in this study, its expression was not induced by ER stress linked to Alzheimer’s disease^27^.

We identified several enhancer elements for ER stress in the 5′ upstream region of the transmembrane E3 ligases by *in silico* search (RNF130, RNF149, RNF145, RNF121, RNF19A, RNF170 and RNF180). HRD1 expression is induced by the overexpression of ATF6 and XBP1, because HRD1 has ERSE-I and UPRE consensus sequences in its 5′ upstream region^14^,^28^. However, no E3 gene, besides HRD1, was upregulated in response to ER stress. On the other hand, although CGRRF1 and RNF19B or RNFT1 responded to ATF6 or XBP1, respectively, no consensus sequence for ER stress seems to exist in these promoters. Because various types of enhancer elements, which are slightly different from the consensus sequences, responsible for ER stress have been identified^15^,^28^, it is difficult to predict the responsiveness to ER stress from only these enhancer elements.

So far, it has remained unknown whether the majority of transmembrane E3 ligases are involved in unfolded protein degradation as components of the ERAD machinery. We identified RNFT1 as a novel candidate for ERAD E3, on the basis of responsiveness and resistance to ER stress. RNFT1 showed RING-dependent E3 activity, whereby it suppressed ER stress-induced cell death in a stress-specific manner as in the case for HRD1^13^. To date, several of the E3s identified in this study, including AMFR/gp78^29^, RNF103/KF1^30^ and RNF139/TRC8^31^, have been reported to be involved in ERAD. A common feature of these genes, including RNFT1, is that they encode for multiple transmembrane domains (4 or more; Table 2 and Supplementary Fig. S1). Furthermore, we demonstrated that RNF145 has ER stress-specific resistance similar to RNFT1 (Supplementary Fig. S7), although its expression was not induced by ER stress (Supplementary Fig. S6a). We and others have demonstrated that the multi-transmembrane domains of HRD1 are necessary to export ERAD substrates to the cytosol in the ERAD pathway^32^,^33^. Based on these findings, we propose that the multi-transmembrane domains of E3s play a critical role in exporting proteins from the ER to the cytosol in the ERAD pathway. On the other hand, ER stress responsiveness is not always necessary for ERAD E3s, since gene expression of RNF145, as well as AMFR, RNF103 and RNF139, is not induced by ER stress. However, ER localisation and ER stress resistance seem to be more effective indicators, particularly if other genes that were not induced by ER stress as well as RNF145 can be tested experimentally using cell lines in which they are stably overexpressed.

In this study, RNF185 also prevented ER stress-induced cell death. It has been reported that RNF185 can be involved in the ubiquitination and degradation of the cystic fibrosis transmembrane conductance regulator (CFTR), a common ERAD substrate^34^. On the other hand, RNF185 also localises to the mitochondrial outer membrane and catalyses K63-linked polyubiquitination of the Bcl-2 family protein BNIP1^35^, including p62 recruitment leading to mitophagy. Proteins conjugated to K48-linked ubiquitin chains are targeted to the proteasome for degradation, whereas proteins conjugated to K63-ubiquitin chains are involved in endosomal trafficking to the lysosome, intracellular signalling or DNA repair^36^,^37^. It was recently reported that RNF152, a single transmembrane protein, localizes to lysosomes and regulates mTORC1 signalling by mediating K63-linked polyubiquitination^38^. Taken together, these findings suggest that transmembrane E3 ligases that localise to organelles other than the ER can potentially catalyse K63-linked polyubiquitination. In addition, the K63-linkage-forming E3s with transmembranes seem to contain either one or two transmembrane domains, in contrast to the ER-resident K48-linkage-forming E3s with multi-transmembrane domains.

In the present study, we identified E3 ligases with transmembrane domains and demonstrated their possible involvement in ERAD. Furthermore, we found several tissue-specific/selective E3 genes, whilst the others were all expressed in a wide range of tissues. Thus, it is possible that some E3s have developmental stage-specific roles. However, the substrates and physiological/pathological functions of the majority of E3s identified in this study remain to be elucidated. Hence, to understand their roles (whether ERAD or membrane trafficking), it is critical to determine the substrates, cellular localisation and ubiquitin chain pattern of those E3s. Furthermore, their tissue specific or developmental stage-specific roles should be also addressed in future studies.

## Methods

### Animals

All animal experiments were performed in accordance with the NIH Guidelines for the care and use of laboratory animals and were approved by the Committee of Animal Experimentation, Hiroshima University. The mice were maintained in a room at 23 °C under a constant day–night rhythm and given food and water ad libitum.

### Cell culture

Human cervical carcinoma HeLa, mouse neuroblastoma Neuro2a (N2a) and monkey kidney COS-1 cells were maintained in Dulbecco’s modified Eagle’s medium (DMEM; Sigma-Aldrich, St. Louis, MO) with 10% (v/v) heat-inactivated foetal calf serum at 37°C in 5% CO_2_, 95% humidified air atmosphere.

### Plasmids and stable cell lines

Human E3 ligases were amplified from cDNA of Tm-treated HeLa cells using gene-specific primers (forward primers with the 5′-CACC overhang and reverse primers without stop codons; Supplementary Table S2) and cloned into the pENTR Directional TOPO vector (entry clone; Life Technologies, Carlsbad, CA). The cloned sequences were then transferred into the pcDNA6.2/V5-DEST vector, including the V5 epitope tag at the C-terminal of the insert sequences (Life Technologies), using Gateway LR Clonase II Enzyme mix (Life Technologies). The CS mutants were constructed by PCR using the overlapping method with the QuikChange Site-Directed Mutagenesis Kit (Agilent Technologies, Santa Clara, CA).

For stable cell lines, entry clone products and the pENTR5′/CMVp vector were recombined into the pLenti 6.4/R4R2/V5-DEST vector using Gateway LR Clonase II Plus enzyme mix (Life Technologies). To produce lentivirus carrying V5-tagged E3s, the pLenti-based expression vectors and the ViraPower Packaging mix (Life Technologies) were cotransfected into the 293FT cell line. The virus-containing supernatant was harvested and transduced into COS-1 and N2a cells. Cells stably expressing E3s were selected with 5 μg/ml blasticidin S hydrochloride (WAKO Pure Chemical Industries, Osaka, Japan).

### Databases and predication for protein

Public protein databases (RefSeq and Ensembl) were searched for typical RING-finger motifs (C3H2C3 or C3HC4; C-X_2_-C-X_9-39_-C-X_1-3_-H-X_2-3_-C/H-X_2_-C-X_9-39_-C-X_2_-C). The prediction of transmembrane helices in proteins was performed by the TMHMM Server v. 2.0.

### Analysis of mRNA levels for human and mouse tissues

Human tissue total RNAs (Human Total RNA Master Panel II) were purchased from TaKaRa Biotechnology (Otsu, Japan). The total RNA sources were pooled from one to 10 persons. Mouse tissue total RNAs were extracted from 6-week-old male C57BL/6 mice using ISOGEN (Nippon Gene, Toyama, Japan). Reverse transcription was performed with ReverTra Ace (TOYOBO, Osaka, Japan). The reverse-transcribed cDNA was measured by TaqMan-based real-time PCR assay using the THUNDERBIRD Probe qPCR Mix (TOYOBO) on a LightCycler 480 Instrument II real-time PCR system (Roche diagnostics, Basel, Switzerland). TaqMan primer and probe sets for human and mouse (formally called PrimeTime qPCR Assays for the mouse sets; Supplementary Table S3) were purchased from Life Technologies and Integrated DNA Technologies (IDT; Coralville, IA), respectively.

### Analysis of mRNA levels for culture cells

HeLa cells were treated with Tg (1 μM) and Tm (3 μg/ml). HeLa pcDNA vectors containing human ATF6α (encoding N-terminal 93 amino acid residues) or human XBP1s (encoding spliced form) were transfected into HeLa cells using ScreenFect A (WAKO Pure Chemical Industries). Total RNAs from the cells were extracted using ISOGEN. The reverse-transcribed cDNA was measured by TaqMan-based real-time PCR assay using the delta–delta Ct method.

### Immunocytochemistry

COS-1 cells stably expressing WT-E3s were fixed with 4% paraformaldehyde for 15 min and permeabilized with methanol for 10 min at −20 °C. The cells were then stained with anti-V5 antibody (Life Technologies) and an anti-PDI antibody (Cell signalling Technology, Danvers, MA). Anti-mouse IgG antibody conjugated with Alexa Fluor 488 and anti-rabbit IgG antibody with Alexa Fluor 568 were used for second antibodies (Life Technologies). Fluorescence images were acquired using an Olympus FluoView FV1000 confocal microscope (Tokyo, Japan).

### *In vitro* autoubiquitination assay

V5-tagged human E3s were produced using a TNT-quick coupled transcription/translation system (Promega Corporation, Madison, WI). The reaction mixture was again immunoprecipitated with anti-V5 antibody and analysed via Western blotting using anti-ubiquitin and -V5 antibodies. The reaction products containing V5-tagged E3s were immunoprecipitated with anti-V5 antibody, and then mixed with Recombinant Human GST-Ubiquitin E1 Enzyme (UBE1) Protein (100 ng), Recombinant Human GST-UbcH5c/UBE2D3 Protein (100 ng), and Recombinant Human HA-Ubiquitin Protein (10 μg) purchased from Boston Biochem (Cambridge, MA) in a reaction buffer [100 μl, containing 40 mM Tris-HCl (pH 7.6), 5 mM MgCl_2_, 2 mM ATP and 2 mM dithiothreitol]. The reaction solution was incubated at 37 °C for 60 minutes, immunoprecipitated with the anti-V5 antibody (Life Technologies) and subjected to Western blotting using anti-Multiubiquitin Chains antibody (FK-2; Nippon Biotest Laboratories, Kokubunji, Japan).

### Cell death assay

N2a cells stably expressing WT or ΔR mutants of E3s were incubated with Tm (1 μg/ml) and STS (0.1 μM) for 48 h. The cells were washed with phosphate-buffered saline (PBS) and then stained with 0.1% crystal violet (WAKO), and the wells were washed with PBS. The dye was eluted with water containing 0.5% sodium dodecyl sulphate, and the optical density was measured at 590 nm.

### Statistics

All data are expressed as mean ± SD statistical evaluation was performed by ANOVA followed by two-tailed Bonferroni correction for multiple comparisons. Each experiment was performed ≥3 times.

## Additional Information

**How to cite this article**: Kaneko, M. *et al.* Genome-wide identification and gene expression profiling of ubiquitin ligases for endoplasmic reticulum protein degradation. *Sci. Rep.* **6**, 30955; doi: 10.1038/srep30955 (2016).

## Supplementary Material

Supplementary Information

## Figures and Tables

**Table 1:** Human ubiquitin ligase genes encoding RING-finger and transmembrane domains (C3H2C3 type).

Gene Name	Synonym	Upstream motif		Transmembrane	Protein length
RNF130	G1RZFP, GOLIATH, GP	−315	UPRE	2	419
RNF150	KIAA1214			2	438
RNF149	FLJ90504	−2586	ERSE-I	1	400
RNF133				1	376
RNF128	FLJ23516, GRAIL			1	428
RNF122	FLJ12526			1	155
RNF24	G1L			1	148
RNF13	RZF			1	381
RNF167	DKFZP566H073			1	350
ZNRF4	nixin, RNF204, sperizin, spzn, Ssrzf1			1	429
RNF43^†^	DKFZp781H0392, FLJ20315, URCC			2	783
ZNRF3^†^	BK747E2.3, FLJ22057, KIAA1133, RNF203			1	936
SYVN1^‡^	DER3, HRD1	−506−2303	ERSE-I	6	617
AMFR	gp78, RNF45			5	643
RNF175^§^	FLJ34190			5	328
RNF121^§^	FLJ11099	−2372	UPRE	6	327
RNF145^‡^	FLJ31951	−1258	ERSE-II	12	663
RNF139^‡^	HRCA1, RCA1, TRC8			12	664
RNF103	hkf-1, KF1			4	685

These lists are aligned with the relatedness from a multiple sequence alignment by CLUSTALW. The same gene families are grouped into the same symbol based on the TreeFam.

**Table 2:** Human ubiquitin ligase genes encoding RING-finger and transmembrane domains (C3HC4 type).

Gene Name	Synonym	Upstream motif		Transmembrane	Protein length
RNF19A	DKFZp566B1346, dorfin	−428	UPRE-r	2	838
RNF19B	FLJ90005			2	732
RNF5^†^	G16, NG2, RING5, RMA1			2	180
RNF185^†^	FLJ38628			2	192
RNF170	ADSA, DKFZP564A022	−627	UPRE	3	258
RNF186^‡^	FLJ20225			2	227
RNF152^‡^	FLJ39176			1	203
RNF183	MGC4734			1	192
RNF182^‡^	MGC33993			2	247
TRIM59^§^	Mrf1, RNF104, TSBF1			1	403
TRIM13^§^	DLEU5, Leu5, RNF77			1	407
BFAR	BAR, RNF47			3	450
RNF180		−4801	ERSE-II	1	592
RNFT1	PTD016			5	435
RNF26	MGC2642			4	433
CGRRF1	CGR19, RNF197			1	332
MUL1	FLJ12875, GIDE, MAPL, MULAN, RNF218			2	352
RNF217	dJ84N20.1, MGC26996			1	542

These lists are aligned with the relatedness from a multiple sequence alignment by CLUSTALW. The same gene families are grouped into the same symbol based on the TreeFam.
