# Supplementary material for: Genome-wide identification and gene expression profiling of ubiquitin ligases for endoplasmic reticulum protein degradation
Source: Sci Rep. 2016 Aug 3;6:30955. doi: 10.1038/srep30955 (PMC4971459; doi:10.1038/srep30955)
Supplement: Supplementary Information [file srep30955-s1.pdf]

Genome-wide identification and gene expression profiling of ubiquitin ligases for endoplasmic reticulum protein degradation

Masayuki Kaneko, Ikuko Iwase, Yuki Yamasaki, Tomoko Takai, Yan Wu, Soshi Kanemoto, Koji Matsuhisa, Rie Asada, Yasunobu Okuma, Takeshi Watanabe, Kazunori Imaizumi, Yausyuki Nomura

SUPPLEMENTARY INFORMATION

a

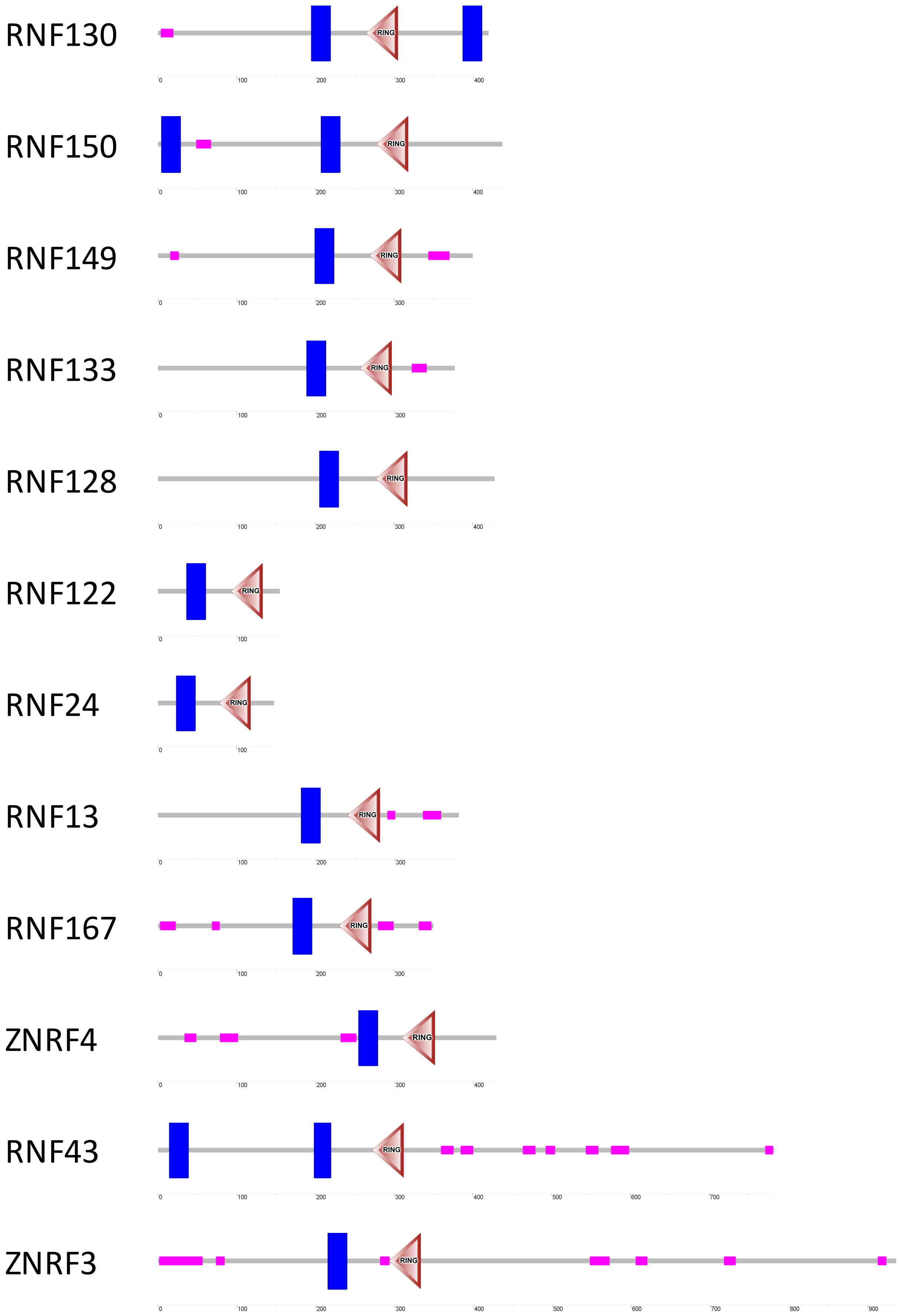
RNF130
RING
RNF150
RING
RNF149
RING
RNF133
RING
RNF128
RING
RNF122
RING
RNF24
RING
RNF13
RING
RNF167
RING
ZNRF4
RING
RNF43
RING
ZNRF3
RING

a

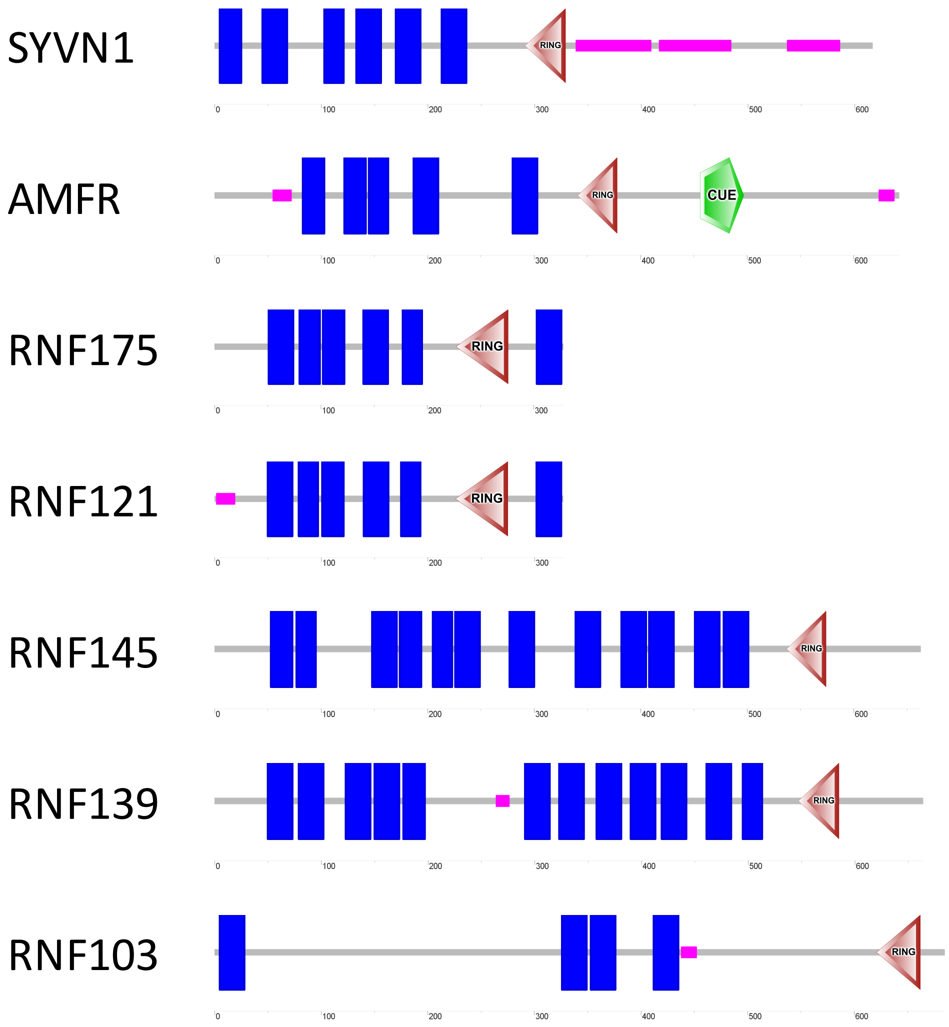
SYVN1
RING
AMFR
RING
CUE
RNF175
RING
RNF121
RING
RNF145
RING
RNF139
RING
RNF103
RING
0
100
200
300
400
500
600

b

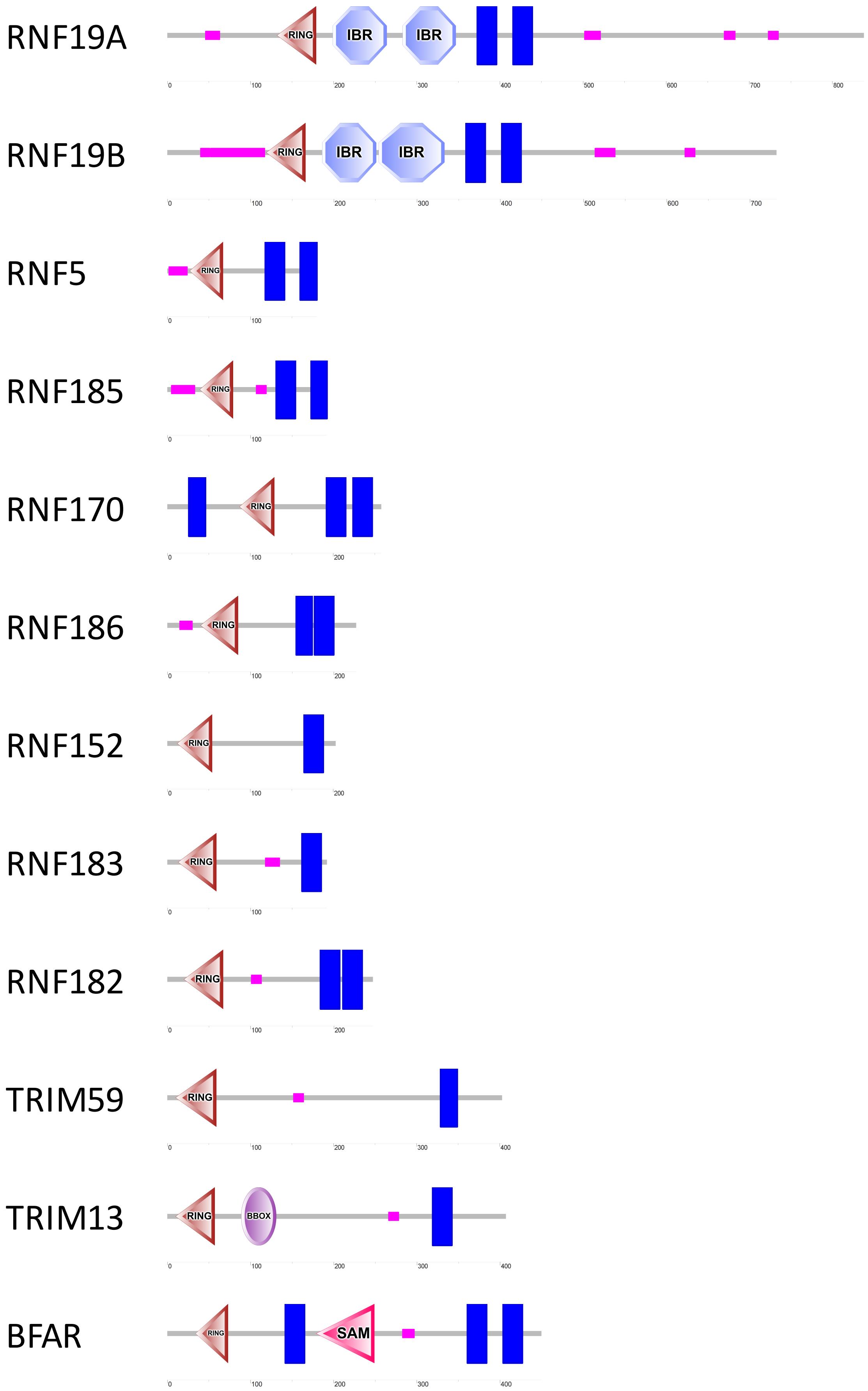
RNF19A
RING
IBR
IBR
RNF19B
RING
IBR
IBR
RNF5
RING
RNF185
RING
RNF170
RING
RNF186
RING
RNF152
RING
RNF183
RING
RNF182
RING
TRIM59
RING
TRIM13
RING
BBOX
BFAR
RING
SAM

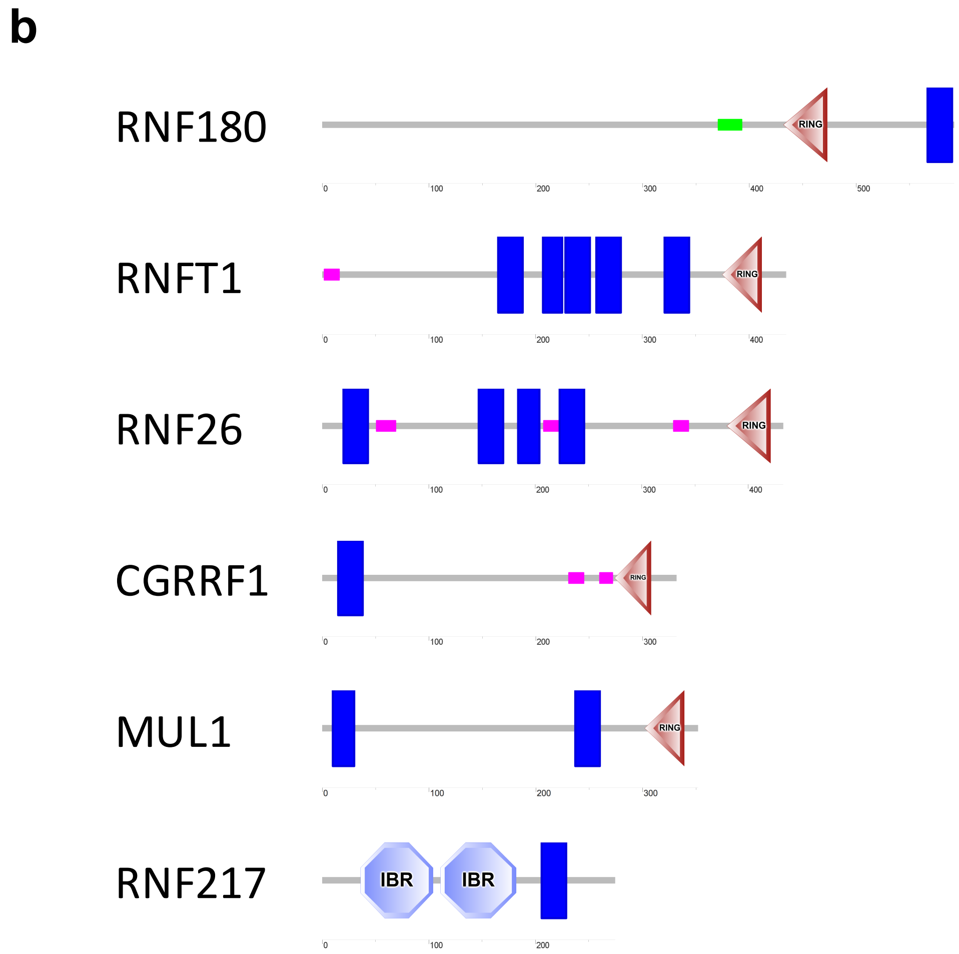

Supplementary Fig. S1. Schematic diagrams of predicted domains for E3 ligases. (**a**) C3H2C3-RING E3s. (**b**) C3HC4-RING E3s. Schematic diagrams of predicted domains for E3 ligases were made with the SMART program. The *blue* box indicates transmembrane helix region, as detected by the TMHMM v2.0 program. The bold line in *pink* represents low complexity sequence. The *red* triangle is the RING-finger domain, and the CUE domain in AMFR is an ubiquitin-binding motif. The IBR (in-between ring fingers) domain in RNF19A, RNF19B and RNF217 is often found between pairs of RING fingers and has been called the C6HC domain. The bold line in *yellowish green* in RNF180 represents a coiled-coil region.

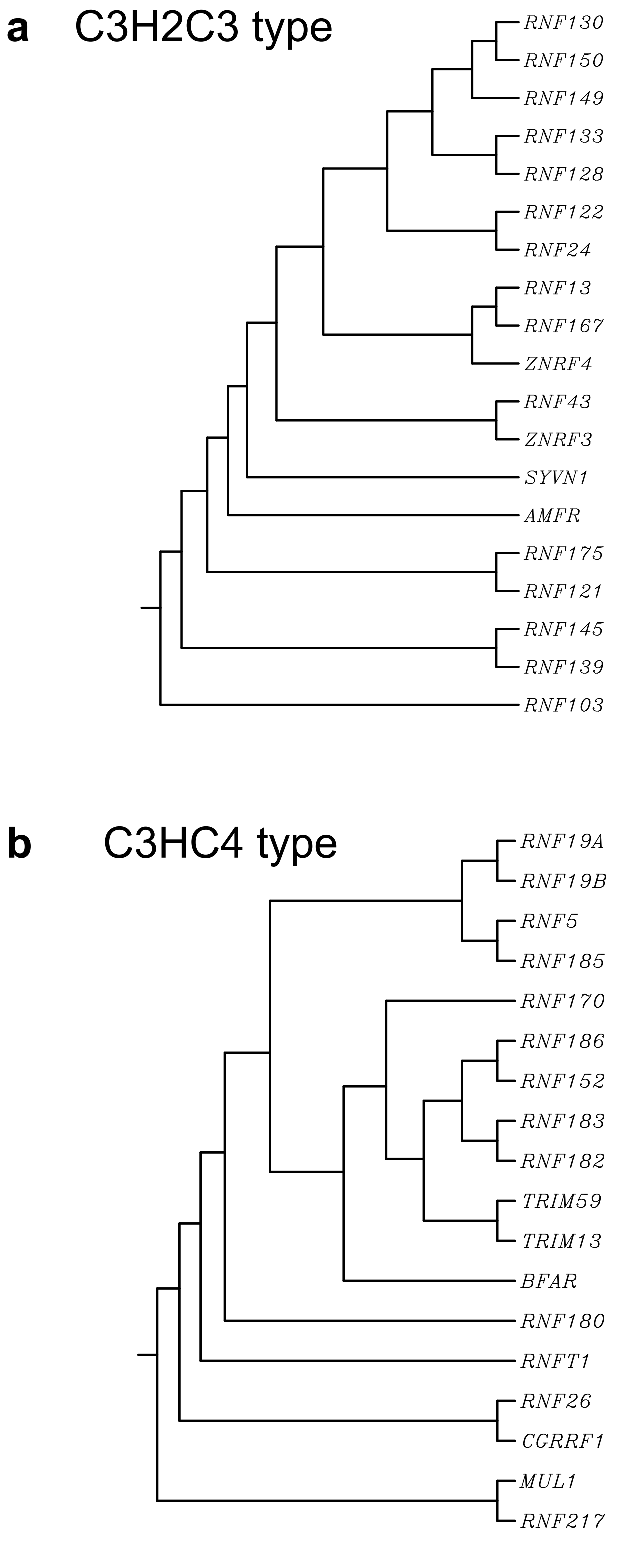

Supplementary Fig. S2. Multiple sequence alignment for E3 ligases. Protein sequences for E3 ligases were aligned with a multiple sequence alignment by CLUSTALW (http://www.genome.jp/tools/clustalw/). (**a**) C3H2C3-RING E3s. (**b**) C3HC4-RING E3s.

a RNF130 family

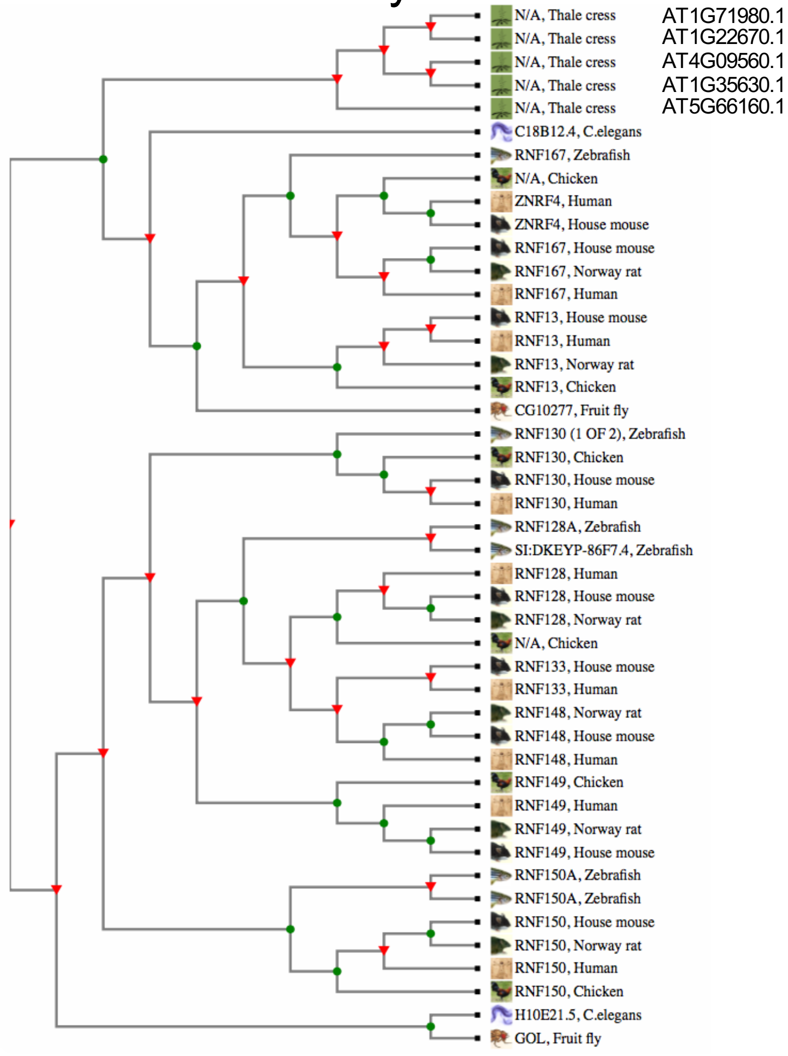

b RNF43 family

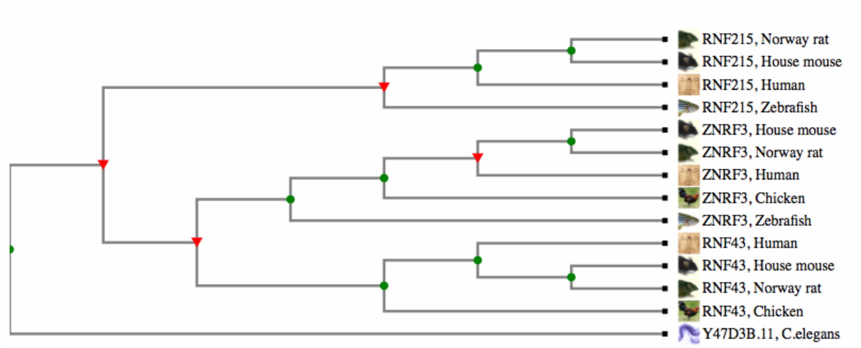

c SYVN1 family

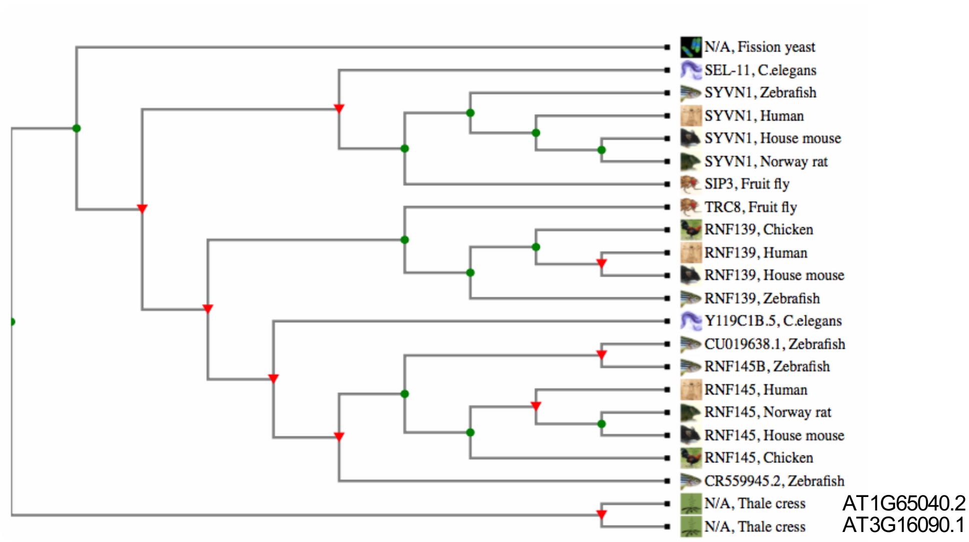

d RNF175 family

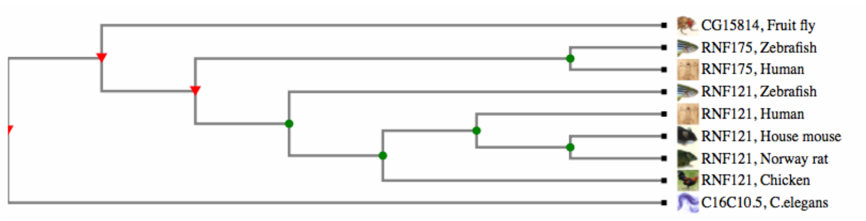

e RNF19A family

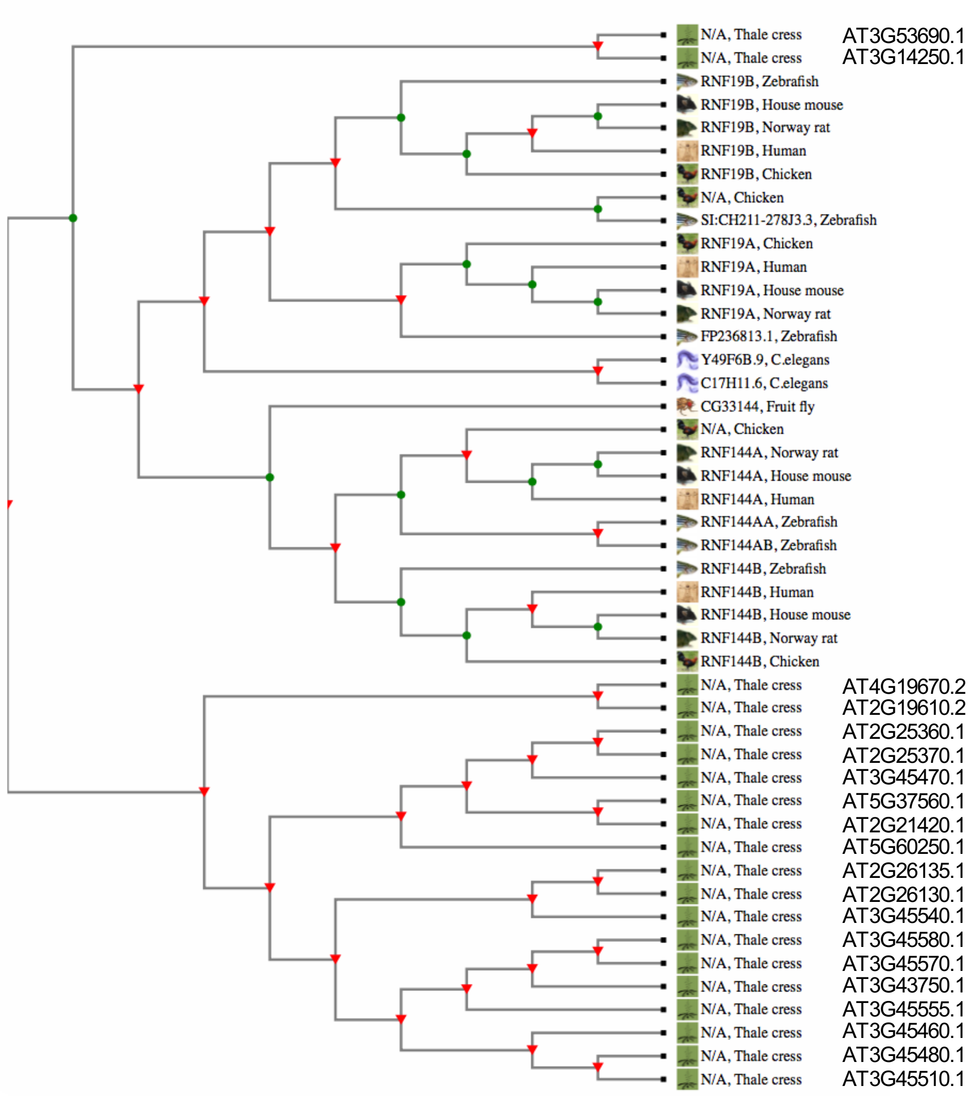

f RNF5 family

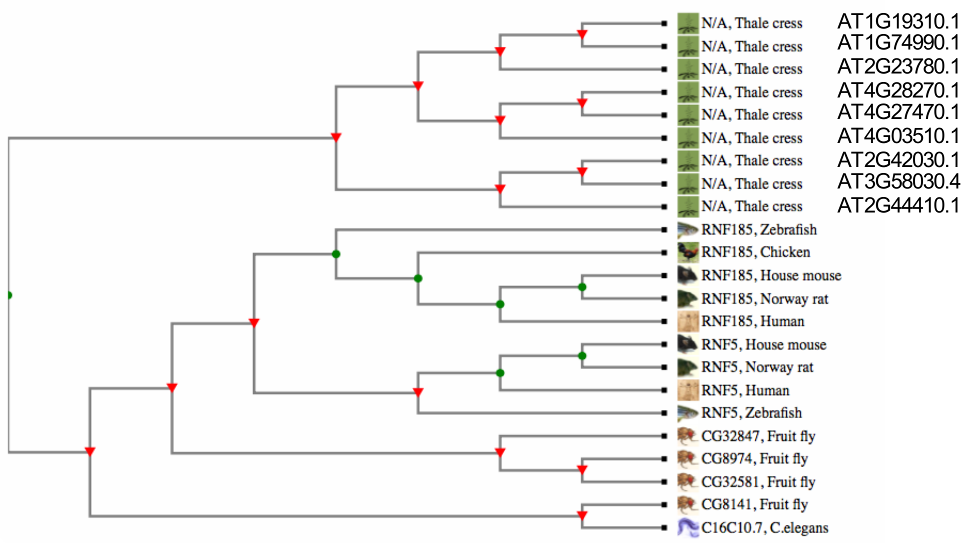

g RNF186 family

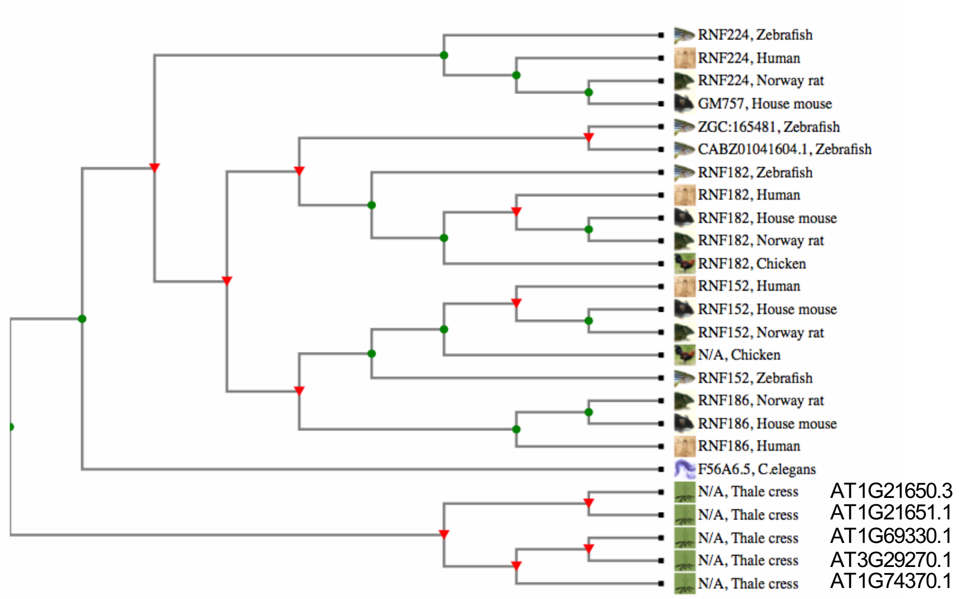

h RNF183 family

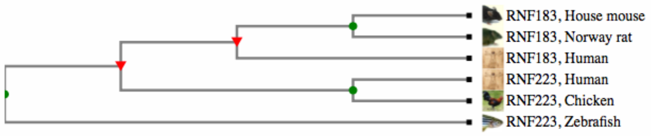

i TRIM59 family

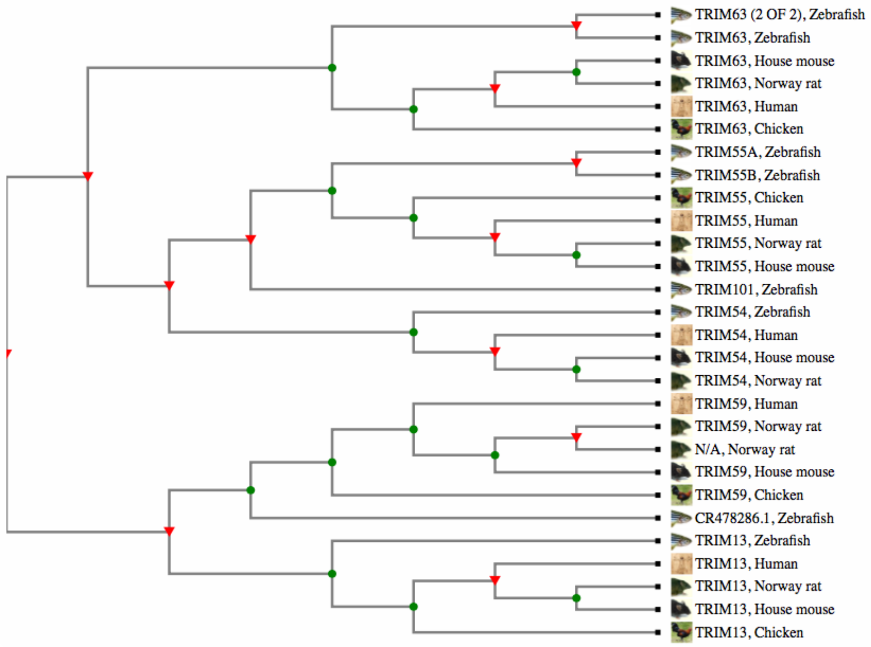

j RNFT1 family

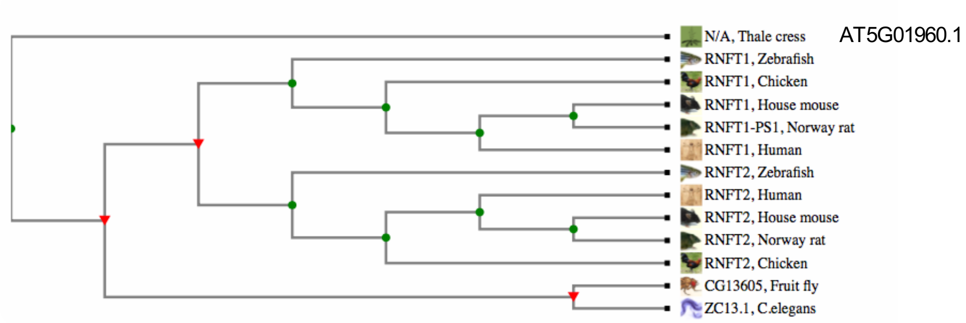

Supplementary Fig. S3. Phylogenetic trees for E3 ligases. Gene trees of E3 ligases were from the TreeFam data base (http://www.treefam.org). TreeFam automatically provides phylogenetic trees for model animal gene families as well as orthologue and paralogue assignments (for more detail, see http://www.treefam.org/help#tabview=tab1). Sequences were aligned using M-Coffee, and TreeBest[1] was used to build a gene tree and reconcile it with the species tree. TreeBest builds five source trees using nj and Phyml[2], and then merges them into one tree while attempting to minimise the number of duplications using a species tree. N/A (not applicable) indicates no gene name but it exists in the species with accession numbers for reference.

SUPPLEMENTARY REFERENCES

1 Ruan, J. *et al.* TreeFam: 2008 Update. *Nucleic Acids Res* **36**, D735-740 (2008).

2 Guindon, S. *et al.* New algorithms and methods to estimate maximum-likelihood phylogenies: assessing the performance of PhyML 3.0. *Syst Biol* **59**, 307-321 (2010).

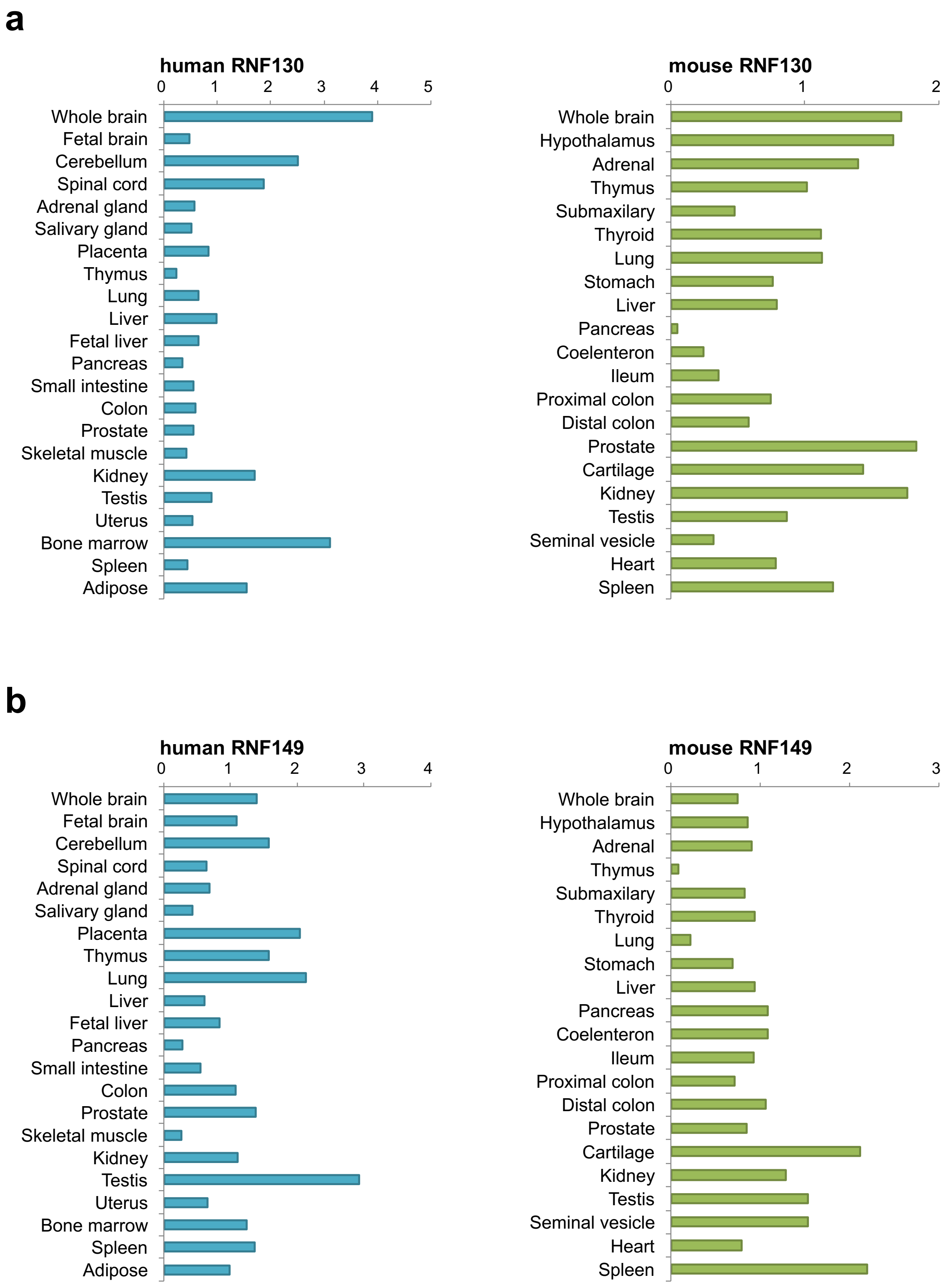
a
human RNF130
0
1
2
3
4
5
Whole brain
Fetal brain
Cerebellum
Spinal cord
Adrenal gland
Salivary gland
Placenta
Thymus
Lung
Liver
Fetal liver
Pancreas
Small intestine
Colon
Prostate
Skeletal muscle
Kidney
Testis
Uterus
Bone marrow
Spleen
Adipose
mouse RNF130
0
1
2
Whole brain
Hypothalamus
Adrenal
Thymus
Submaxilary
Thyroid
Lung
Stomach
Liver
Pancreas
Coelenteron
Ileum
Proximal colon
Distal colon
Prostate
Cartilage
Kidney
Testis
Seminal vesicle
Heart
Spleen
b
human RNF149
0
1
2
3
4
Whole brain
Fetal brain
Cerebellum
Spinal cord
Adrenal gland
Salivary gland
Placenta
Thymus
Lung
Liver
Fetal liver
Pancreas
Small intestine
Colon
Prostate
Skeletal muscle
Kidney
Testis
Uterus
Bone marrow
Spleen
Adipose
mouse RNF149
0
1
2
3
Whole brain
Hypothalamus
Adrenal
Thymus
Submaxilary
Thyroid
Lung
Stomach
Liver
Pancreas
Coelenteron
Ileum
Proximal colon
Distal colon
Prostate
Cartilage
Kidney
Testis
Seminal vesicle
Heart
Spleen

c

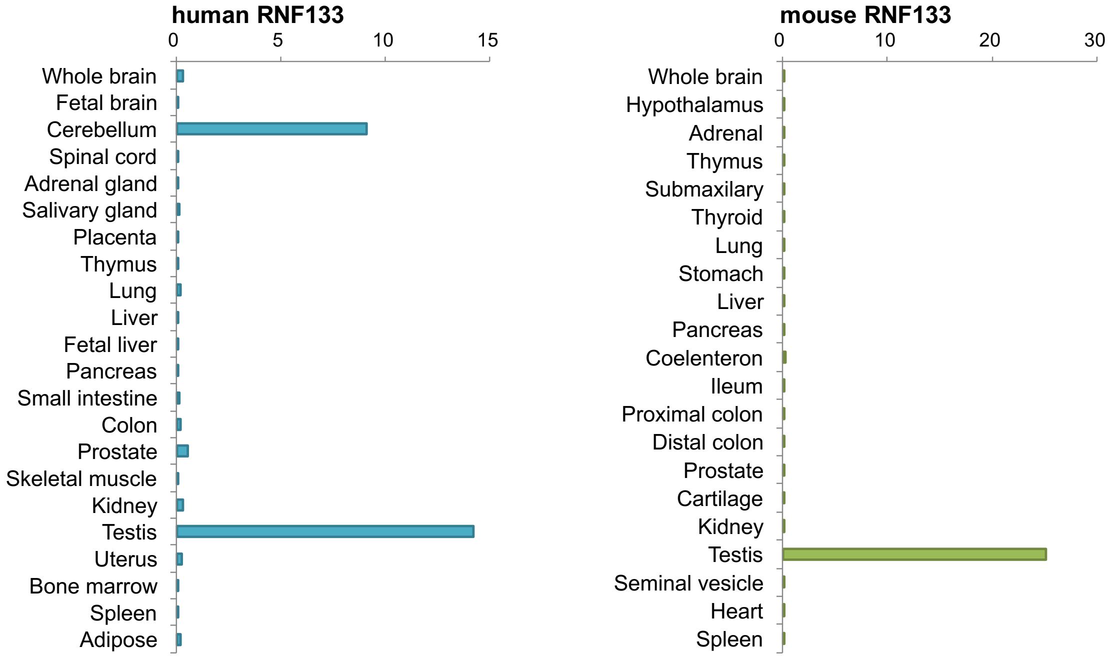
human RNF133
0
5
10
15
Whole brain
Fetal brain
Cerebellum
Spinal cord
Adrenal gland
Salivary gland
Placenta
Thymus
Lung
Liver
Fetal liver
Pancreas
Small intestine
Colon
Prostate
Skeletal muscle
Kidney
Testis
Uterus
Bone marrow
Spleen
Adipose
mouse RNF133
0
10
20
30
Whole brain
Hypothalamus
Adrenal
Thymus
Submaxilary
Thyroid
Lung
Stomach
Liver
Pancreas
Coelenteron
Ileum
Proximal colon
Distal colon
Prostate
Cartilage
Kidney
Testis
Seminal vesicle
Heart
Spleen

d

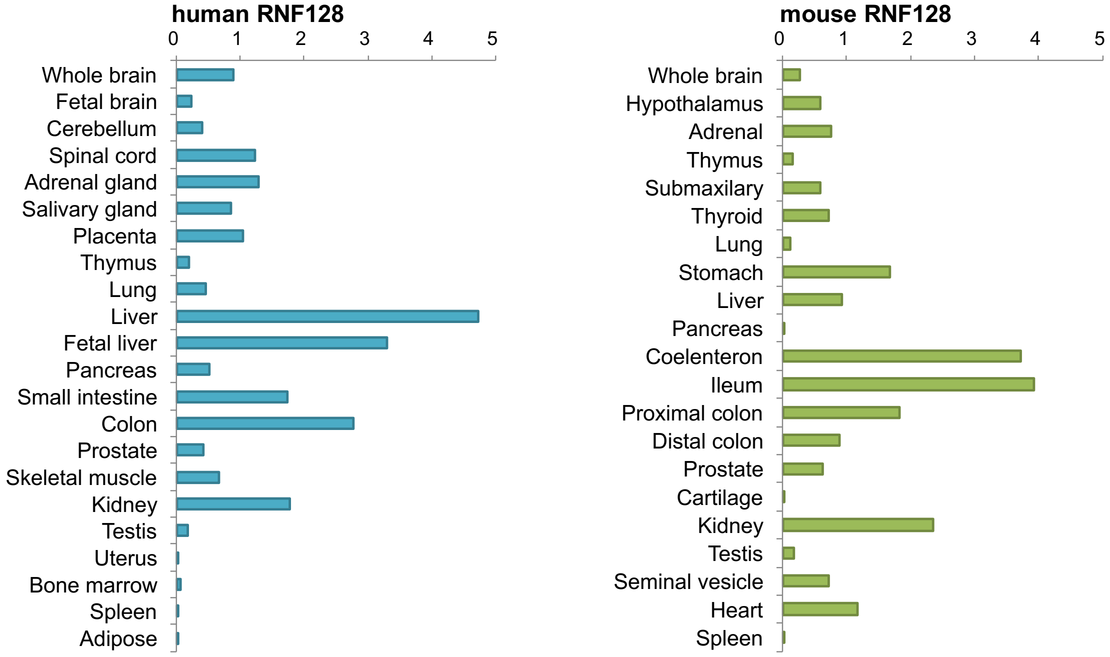
human RNF128
0
1
2
3
4
5
Whole brain
Fetal brain
Cerebellum
Spinal cord
Adrenal gland
Salivary gland
Placenta
Thymus
Lung
Liver
Fetal liver
Pancreas
Small intestine
Colon
Prostate
Skeletal muscle
Kidney
Testis
Uterus
Bone marrow
Spleen
Adipose
mouse RNF128
0
1
2
3
4
5
Whole brain
Hypothalamus
Adrenal
Thymus
Submaxilary
Thyroid
Lung
Stomach
Liver
Pancreas
Coelenteron
Ileum
Proximal colon
Distal colon
Prostate
Cartilage
Kidney
Testis
Seminal vesicle
Heart
Spleen

e

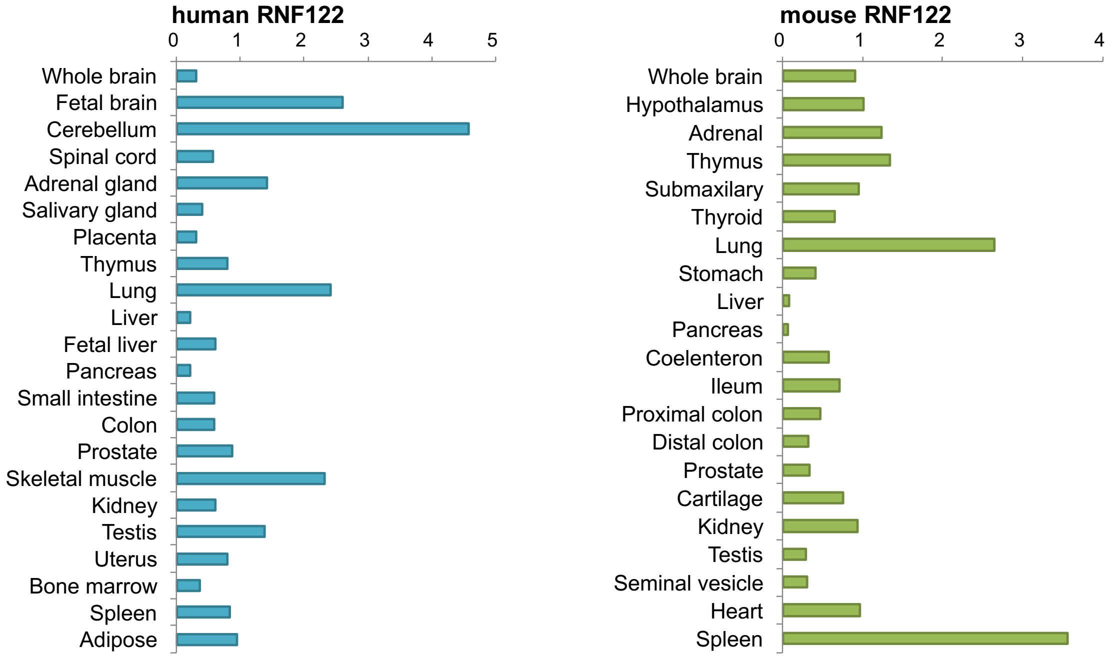
human RNF122
0
1
2
3
4
5
Whole brain
Fetal brain
Cerebellum
Spinal cord
Adrenal gland
Salivary gland
Placenta
Thymus
Lung
Liver
Fetal liver
Pancreas
Small intestine
Colon
Prostate
Skeletal muscle
Kidney
Testis
Uterus
Bone marrow
Spleen
Adipose
mouse RNF122
0
1
2
3
4
Whole brain
Hypothalamus
Adrenal
Thymus
Submaxilary
Thyroid
Lung
Stomach
Liver
Pancreas
Coelenteron
Ileum
Proximal colon
Distal colon
Prostate
Cartilage
Kidney
Testis
Seminal vesicle
Heart
Spleen

f

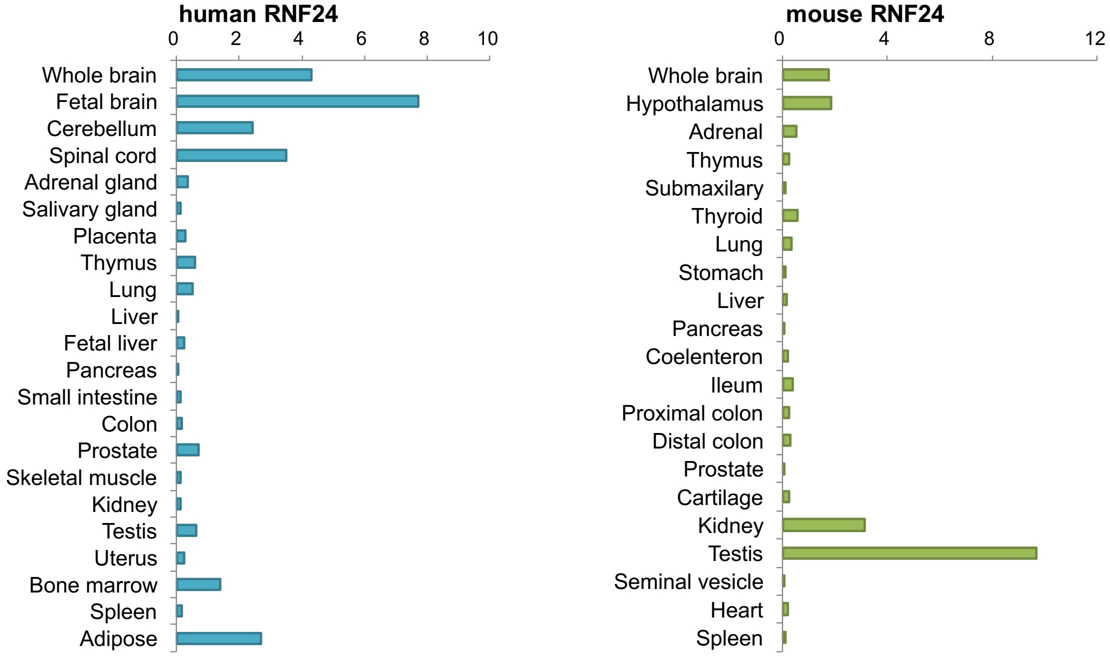
human RNF24
0
2
4
6
8
10
Whole brain
Fetal brain
Cerebellum
Spinal cord
Adrenal gland
Salivary gland
Placenta
Thymus
Lung
Liver
Fetal liver
Pancreas
Small intestine
Colon
Prostate
Skeletal muscle
Kidney
Testis
Uterus
Bone marrow
Spleen
Adipose
mouse RNF24
0
4
8
12
Whole brain
Hypothalamus
Adrenal
Thymus
Submaxilary
Thyroid
Lung
Stomach
Liver
Pancreas
Coelenteron
Ileum
Proximal colon
Distal colon
Prostate
Cartilage
Kidney
Testis
Seminal vesicle
Heart
Spleen

g

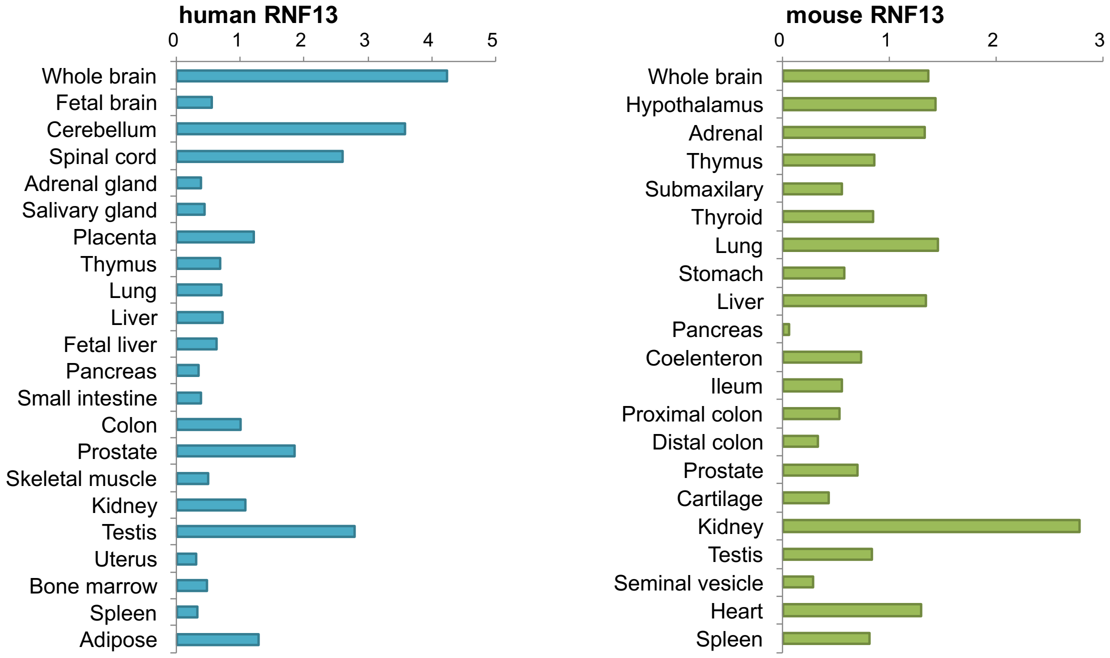
human RNF13
0
1
2
3
4
5
Whole brain
Fetal brain
Cerebellum
Spinal cord
Adrenal gland
Salivary gland
Placenta
Thymus
Lung
Liver
Fetal liver
Pancreas
Small intestine
Colon
Prostate
Skeletal muscle
Kidney
Testis
Uterus
Bone marrow
Spleen
Adipose
mouse RNF13
0
1
2
3
Whole brain
Hypothalamus
Adrenal
Thymus
Submaxilary
Thyroid
Lung
Stomach
Liver
Pancreas
Coelenteron
Ileum
Proximal colon
Distal colon
Prostate
Cartilage
Kidney
Testis
Seminal vesicle
Heart
Spleen

h

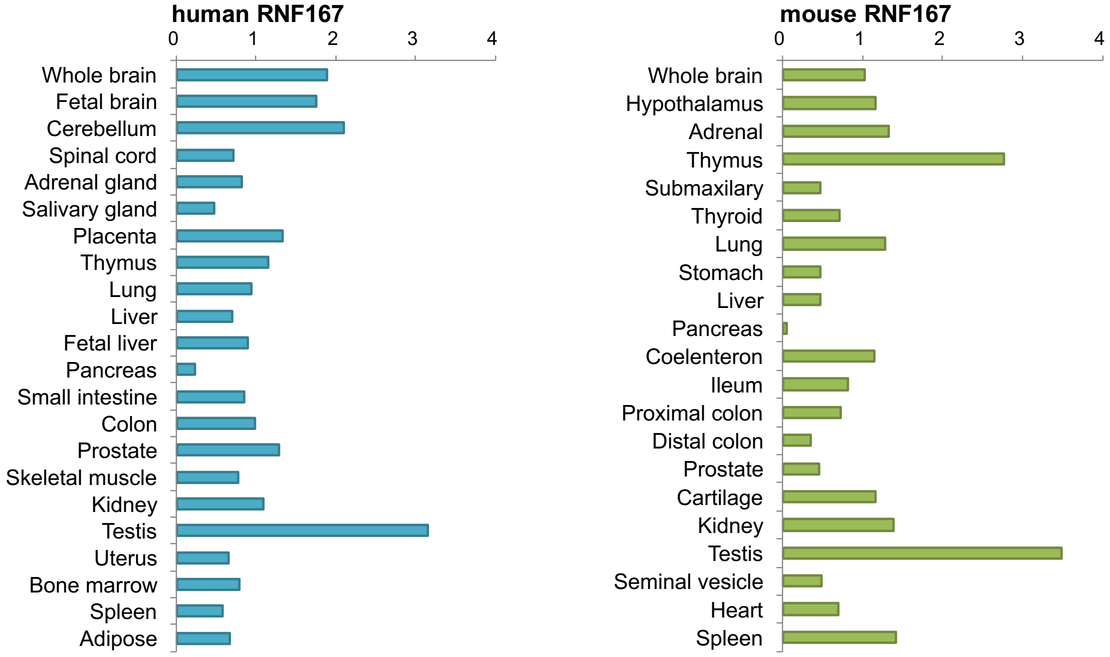
human RNF167
0
1
2
3
4
Whole brain
Fetal brain
Cerebellum
Spinal cord
Adrenal gland
Salivary gland
Placenta
Thymus
Lung
Liver
Fetal liver
Pancreas
Small intestine
Colon
Prostate
Skeletal muscle
Kidney
Testis
Uterus
Bone marrow
Spleen
Adipose
mouse RNF167
0
1
2
3
4
Whole brain
Hypothalamus
Adrenal
Thymus
Submaxilary
Thyroid
Lung
Stomach
Liver
Pancreas
Coelenteron
Ileum
Proximal colon
Distal colon
Prostate
Cartilage
Kidney
Testis
Seminal vesicle
Heart
Spleen

i

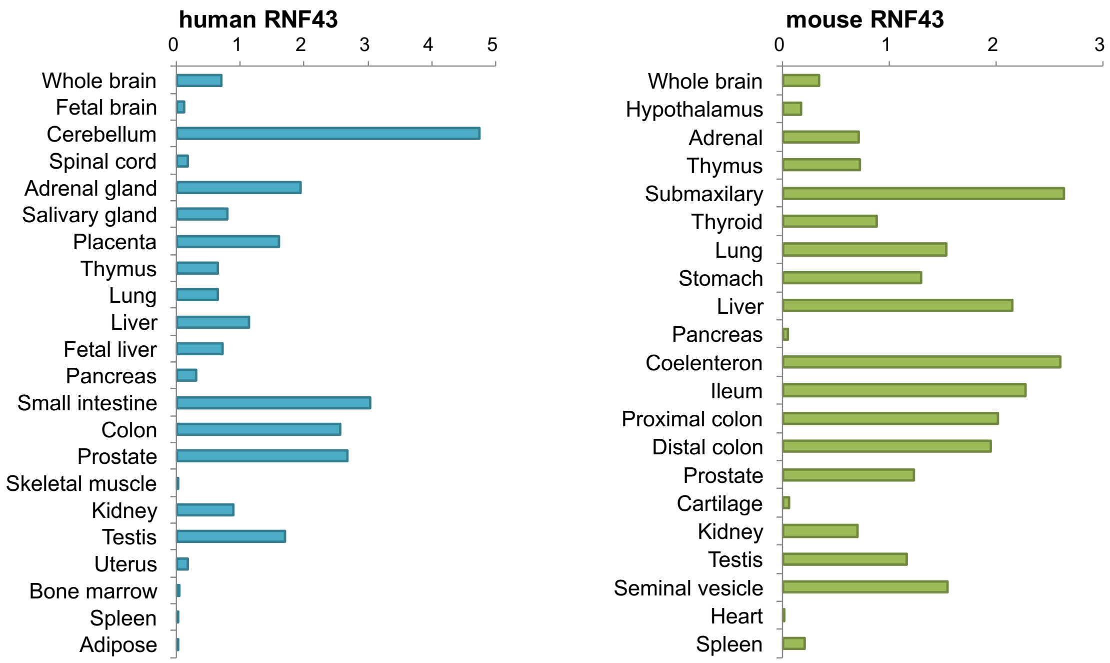
human RNF43
0
1
2
3
4
5
Whole brain
Fetal brain
Cerebellum
Spinal cord
Adrenal gland
Salivary gland
Placenta
Thymus
Lung
Liver
Fetal liver
Pancreas
Small intestine
Colon
Prostate
Skeletal muscle
Kidney
Testis
Uterus
Bone marrow
Spleen
Adipose
mouse RNF43
0
1
2
3
Whole brain
Hypothalamus
Adrenal
Thymus
Submaxilary
Thyroid
Lung
Stomach
Liver
Pancreas
Coelenteron
Ileum
Proximal colon
Distal colon
Prostate
Cartilage
Kidney
Testis
Seminal vesicle
Heart
Spleen

j

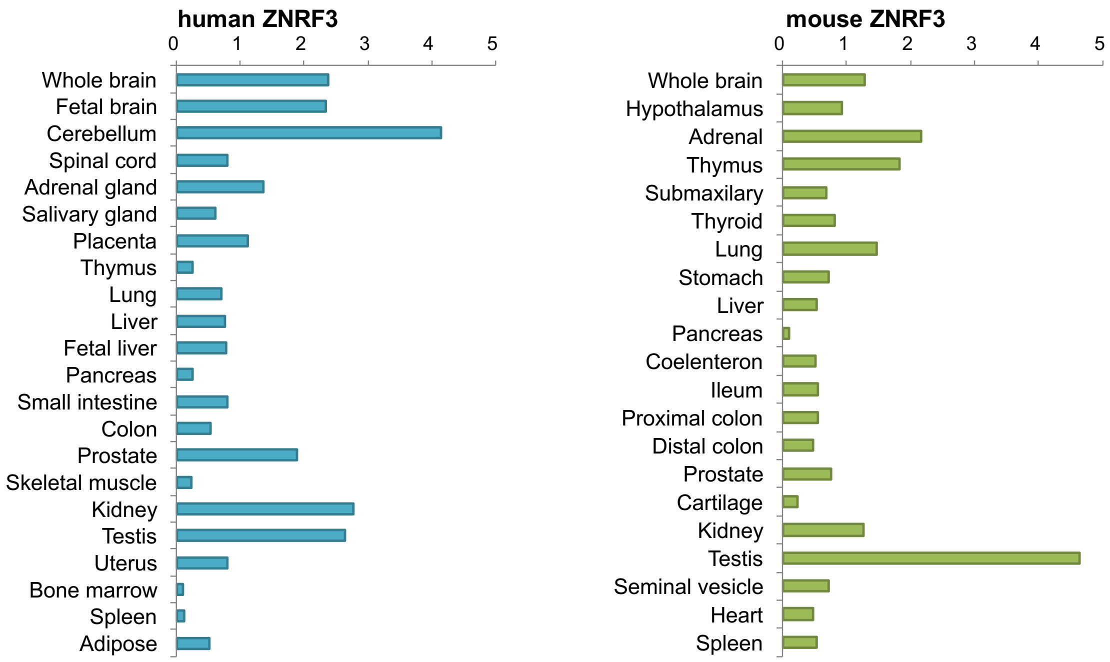
human ZNRF3
0
1
2
3
4
5
Whole brain
Fetal brain
Cerebellum
Spinal cord
Adrenal gland
Salivary gland
Placenta
Thymus
Lung
Liver
Fetal liver
Pancreas
Small intestine
Colon
Prostate
Skeletal muscle
Kidney
Testis
Uterus
Bone marrow
Spleen
Adipose
mouse ZNRF3
0
1
2
3
4
5
Whole brain
Hypothalamus
Adrenal
Thymus
Submaxilary
Thyroid
Lung
Stomach
Liver
Pancreas
Coelenteron
Ileum
Proximal colon
Distal colon
Prostate
Cartilage
Kidney
Testis
Seminal vesicle
Heart
Spleen

k

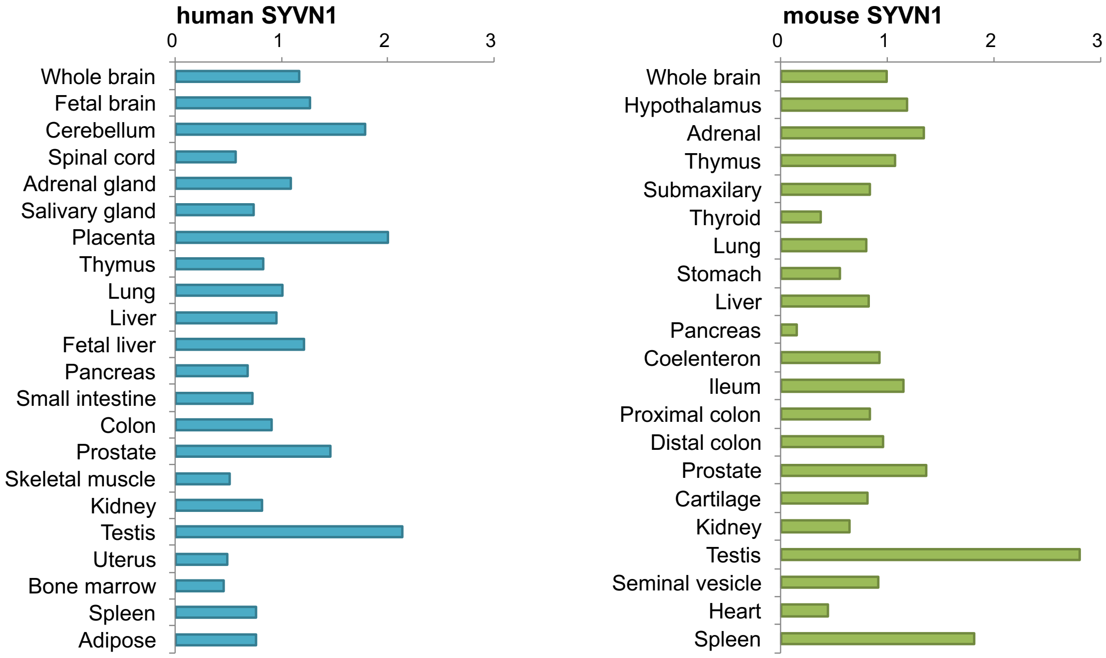

human SYVN1
0
1
2
3
Whole brain
Fetal brain
Cerebellum
Spinal cord
Adrenal gland
Salivary gland
Placenta
Thymus
Lung
Liver
Fetal liver
Pancreas
Small intestine
Colon
Prostate
Skeletal muscle
Kidney
Testis
Uterus
Bone marrow
Spleen
Adipose
mouse SYVN1
0
1
2
3
Whole brain
Hypothalamus
Adrenal
Thymus
Submaxilary
Thyroid
Lung
Stomach
Liver
Pancreas
Coelenteron
Ileum
Proximal colon
Distal colon
Prostate
Cartilage
Kidney
Testis
Seminal vesicle
Heart
Spleen

l

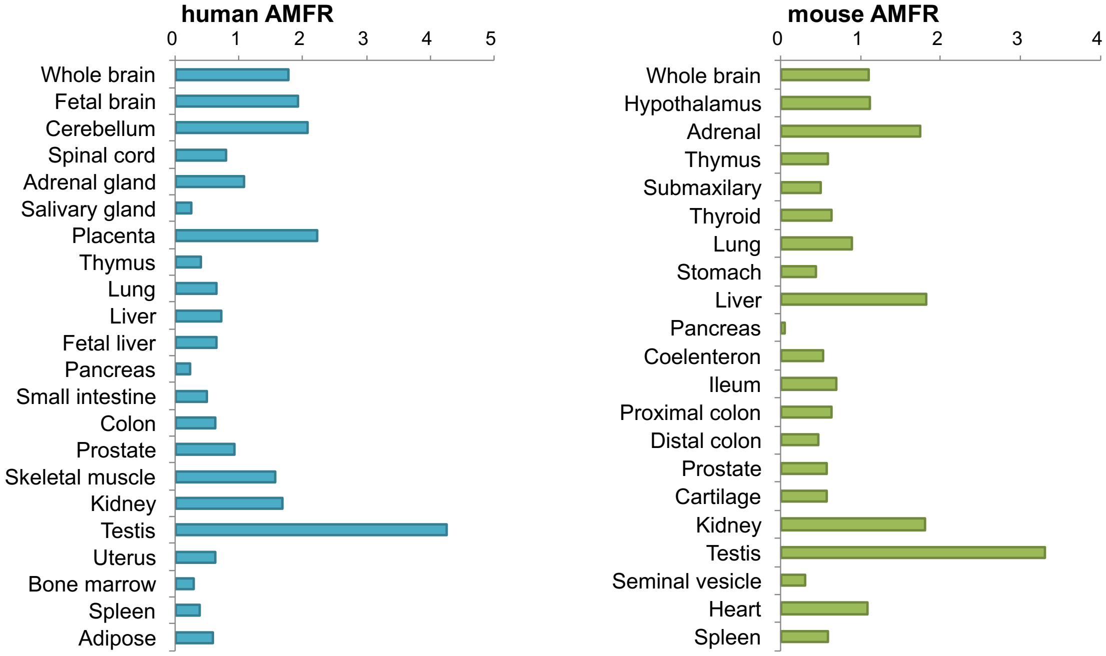

human AMFR
0
1
2
3
4
5
Whole brain
Fetal brain
Cerebellum
Spinal cord
Adrenal gland
Salivary gland
Placenta
Thymus
Lung
Liver
Fetal liver
Pancreas
Small intestine
Colon
Prostate
Skeletal muscle
Kidney
Testis
Uterus
Bone marrow
Spleen
Adipose
mouse AMFR
0
1
2
3
4
Whole brain
Hypothalamus
Adrenal
Thymus
Submaxilary
Thyroid
Lung
Stomach
Liver
Pancreas
Coelenteron
Ileum
Proximal colon
Distal colon
Prostate
Cartilage
Kidney
Testis
Seminal vesicle
Heart
Spleen

m

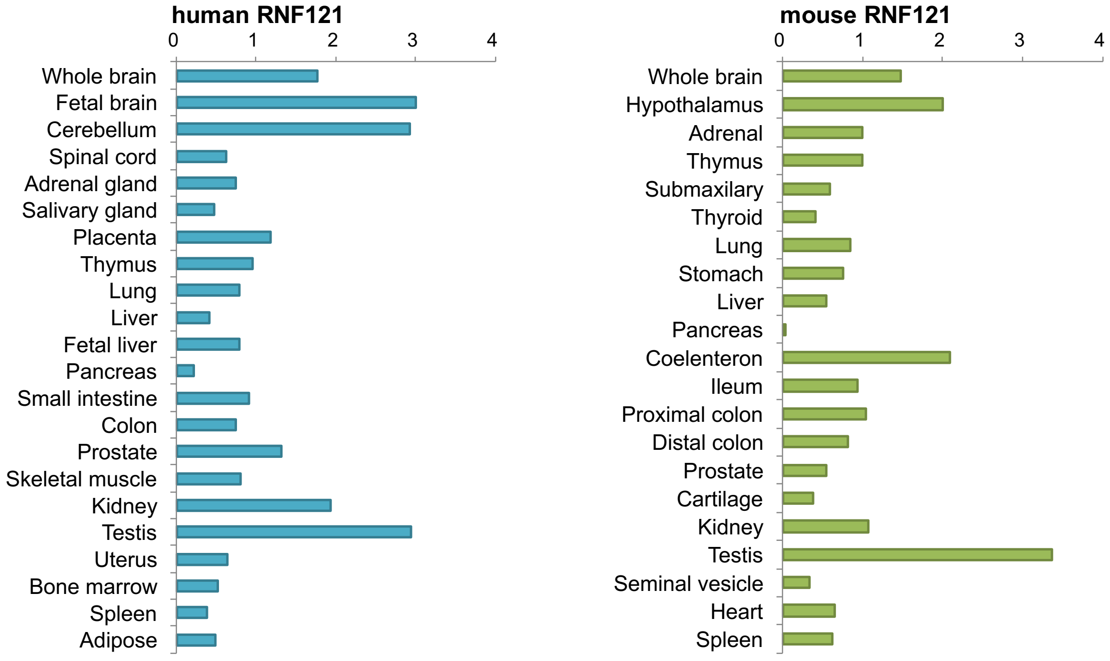
human RNF121
0 1 2 3 4
Whole brain
Fetal brain
Cerebellum
Spinal cord
Adrenal gland
Salivary gland
Placenta
Thymus
Lung
Liver
Fetal liver
Pancreas
Small intestine
Colon
Prostate
Skeletal muscle
Kidney
Testis
Uterus
Bone marrow
Spleen
Adipose
mouse RNF121
0 1 2 3 4
Whole brain
Hypothalamus
Adrenal
Thymus
Submaxilary
Thyroid
Lung
Stomach
Liver
Pancreas
Coelenteron
Ileum
Proximal colon
Distal colon
Prostate
Cartilage
Kidney
Testis
Seminal vesicle
Heart
Spleen

n

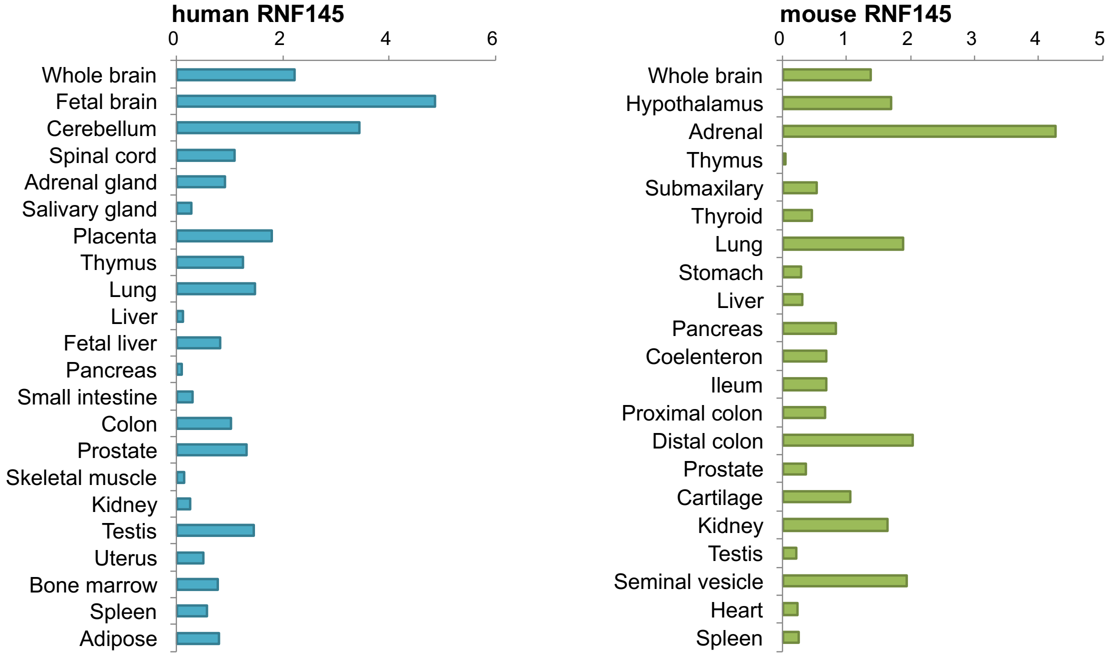
human RNF145
0 2 4 6
Whole brain
Fetal brain
Cerebellum
Spinal cord
Adrenal gland
Salivary gland
Placenta
Thymus
Lung
Liver
Fetal liver
Pancreas
Small intestine
Colon
Prostate
Skeletal muscle
Kidney
Testis
Uterus
Bone marrow
Spleen
Adipose
mouse RNF145
0 1 2 3 4 5
Whole brain
Hypothalamus
Adrenal
Thymus
Submaxilary
Thyroid
Lung
Stomach
Liver
Pancreas
Coelenteron
Ileum
Proximal colon
Distal colon
Prostate
Cartilage
Kidney
Testis
Seminal vesicle
Heart
Spleen

o

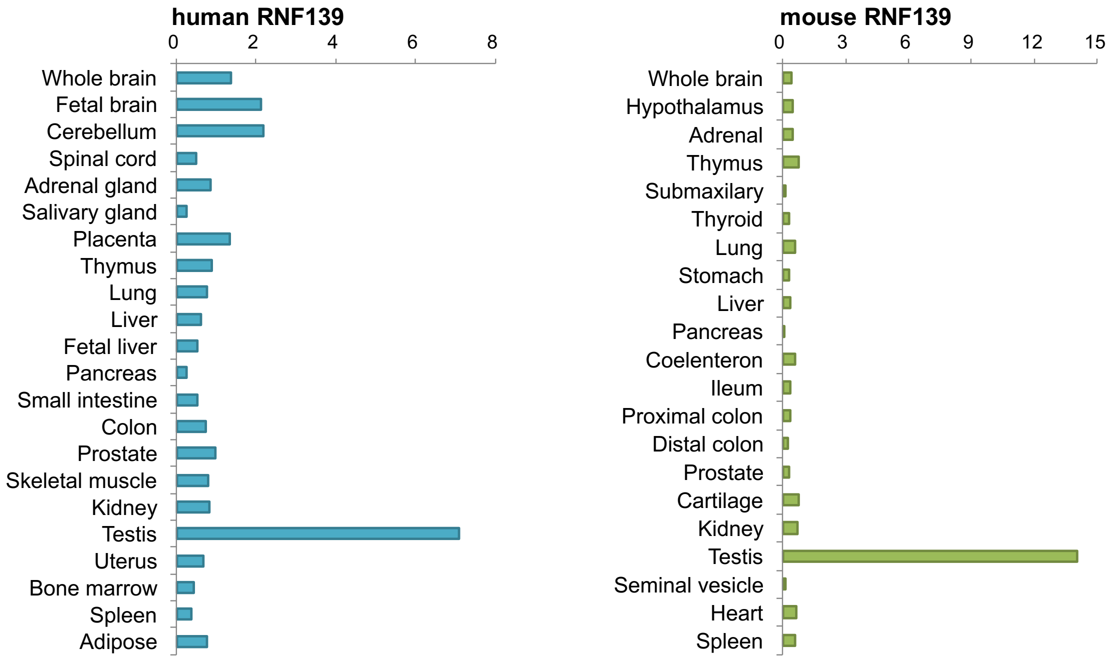

p

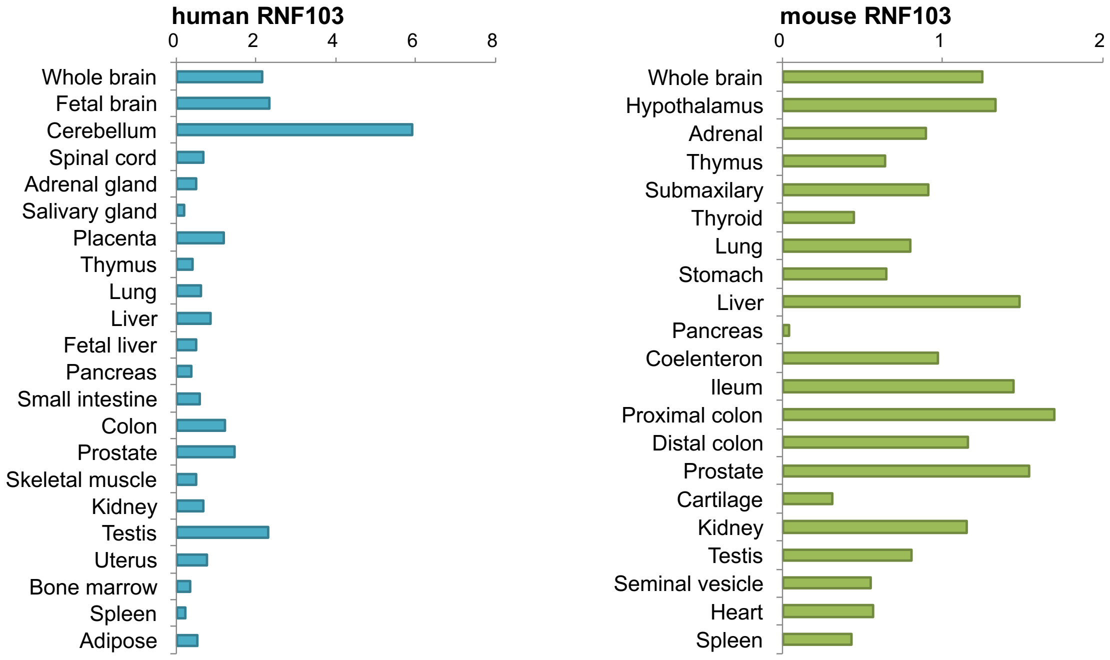

Supplementary Fig. S4. Tissue distribution of E3 ligases (C3H2C3 type) in human and mouse tissues. Total RNAs from 23 human or 22 mouse tissues were reverse-transcribed and measured by TaqMan-based real-time PCR assay using the delta–delta Ct method. Data are normalised to the amount of 18S ribosomal RNA; results are expressed as the fold increase compared with cDNA pools from each tissue.

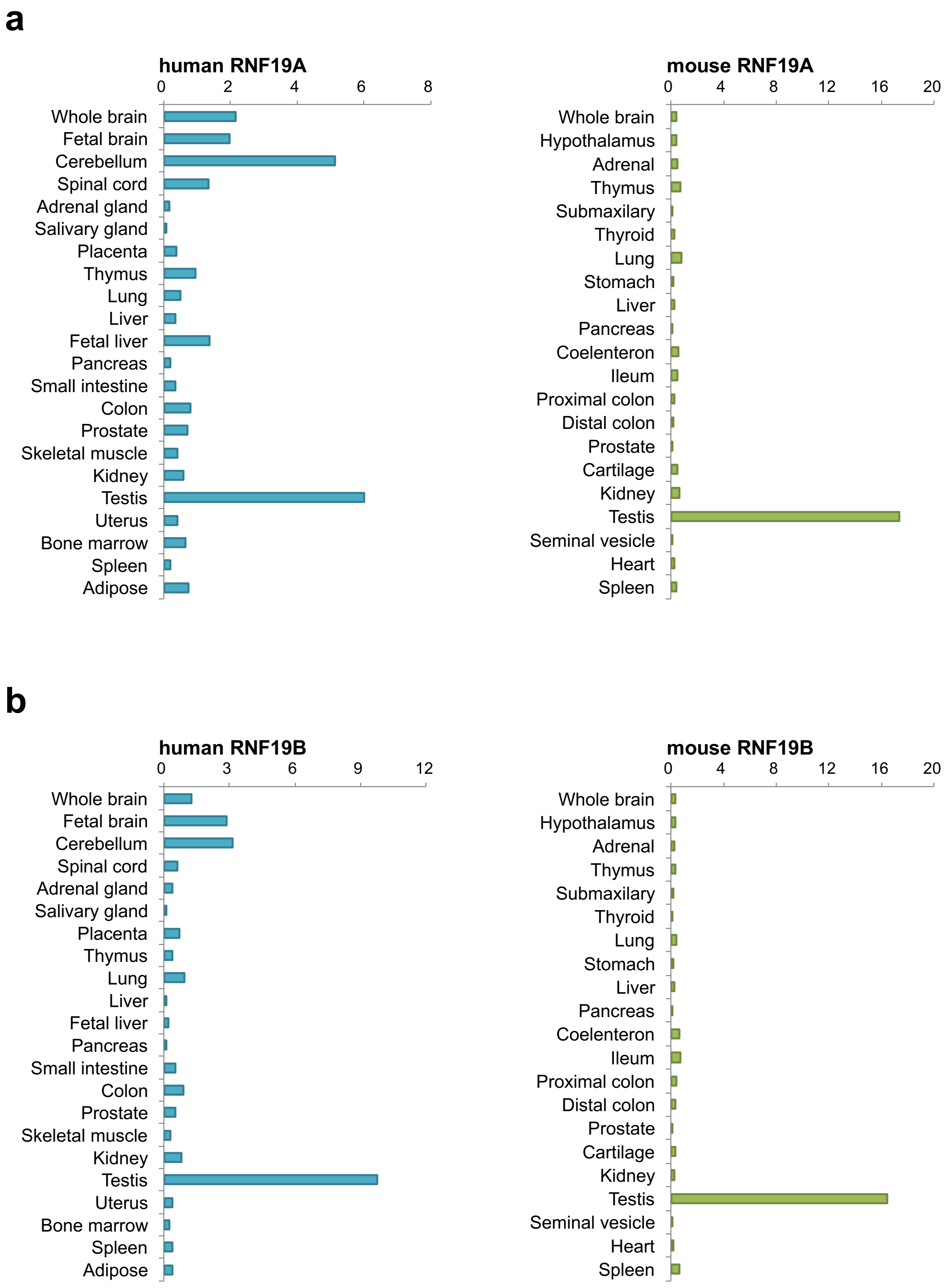
a
human RNF19A
0 2 4 6 8
Whole brain
Fetal brain
Cerebellum
Spinal cord
Adrenal gland
Salivary gland
Placenta
Thymus
Lung
Liver
Fetal liver
Pancreas
Small intestine
Colon
Prostate
Skeletal muscle
Kidney
Testis
Uterus
Bone marrow
Spleen
Adipose
mouse RNF19A
0 4 8 12 16 20
Whole brain
Hypothalamus
Adrenal
Thymus
Submaxilary
Thyroid
Lung
Stomach
Liver
Pancreas
Coelenteron
Ileum
Proximal colon
Distal colon
Prostate
Cartilage
Kidney
Testis
Seminal vesicle
Heart
Spleen
b
human RNF19B
0 3 6 9 12
Whole brain
Fetal brain
Cerebellum
Spinal cord
Adrenal gland
Salivary gland
Placenta
Thymus
Lung
Liver
Fetal liver
Pancreas
Small intestine
Colon
Prostate
Skeletal muscle
Kidney
Testis
Uterus
Bone marrow
Spleen
Adipose
mouse RNF19B
0 4 8 12 16 20
Whole brain
Hypothalamus
Adrenal
Thymus
Submaxilary
Thyroid
Lung
Stomach
Liver
Pancreas
Coelenteron
Ileum
Proximal colon
Distal colon
Prostate
Cartilage
Kidney
Testis
Seminal vesicle
Heart
Spleen

c

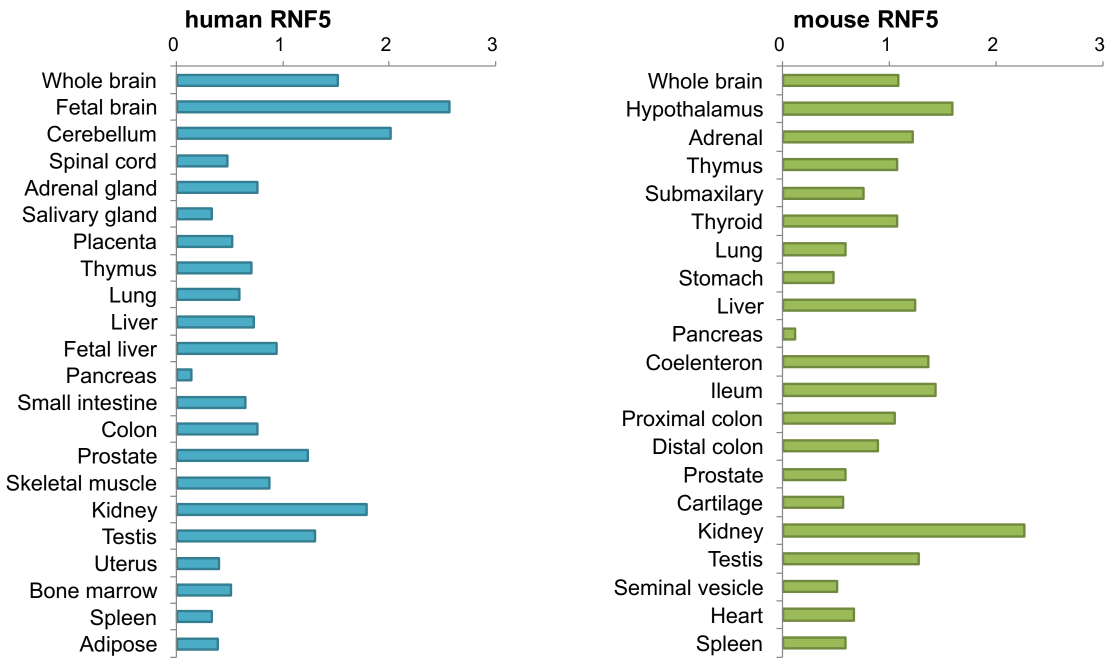
human RNF5
0
1
2
3
Whole brain
Fetal brain
Cerebellum
Spinal cord
Adrenal gland
Salivary gland
Placenta
Thymus
Lung
Liver
Fetal liver
Pancreas
Small intestine
Colon
Prostate
Skeletal muscle
Kidney
Testis
Uterus
Bone marrow
Spleen
Adipose
mouse RNF5
0
1
2
3
Whole brain
Hypothalamus
Adrenal
Thymus
Submaxilary
Thyroid
Lung
Stomach
Liver
Pancreas
Coelenteron
Ileum
Proximal colon
Distal colon
Prostate
Cartilage
Kidney
Testis
Seminal vesicle
Heart
Spleen

d

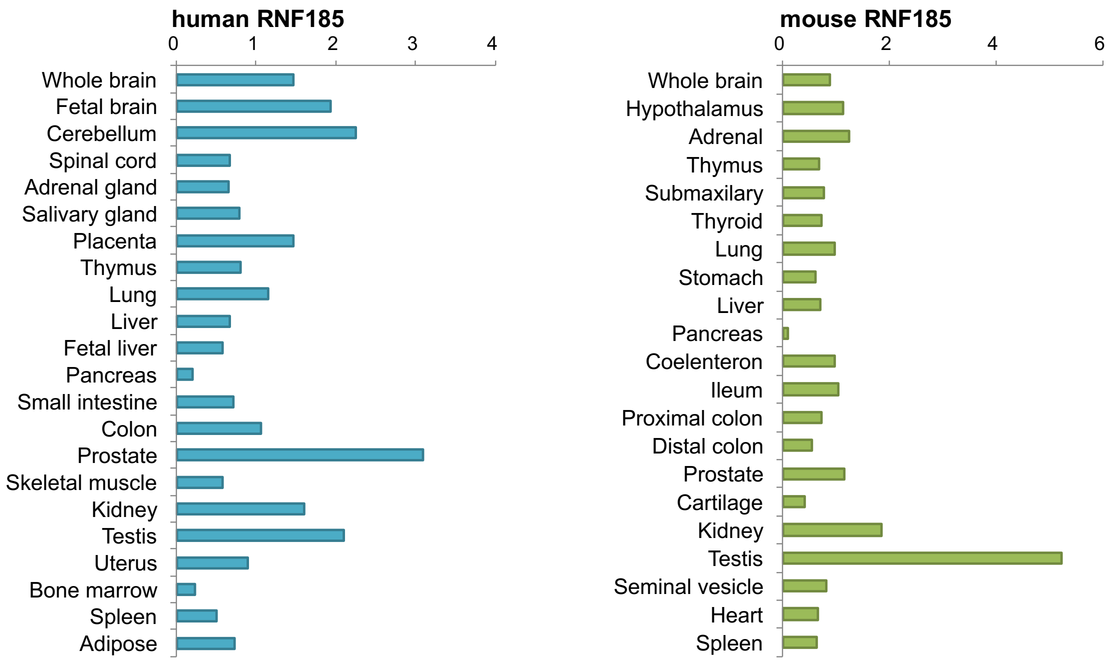
human RNF185
0
1
2
3
4
Whole brain
Fetal brain
Cerebellum
Spinal cord
Adrenal gland
Salivary gland
Placenta
Thymus
Lung
Liver
Fetal liver
Pancreas
Small intestine
Colon
Prostate
Skeletal muscle
Kidney
Testis
Uterus
Bone marrow
Spleen
Adipose
mouse RNF185
0
2
4
6
Whole brain
Hypothalamus
Adrenal
Thymus
Submaxilary
Thyroid
Lung
Stomach
Liver
Pancreas
Coelenteron
Ileum
Proximal colon
Distal colon
Prostate
Cartilage
Kidney
Testis
Seminal vesicle
Heart
Spleen

e

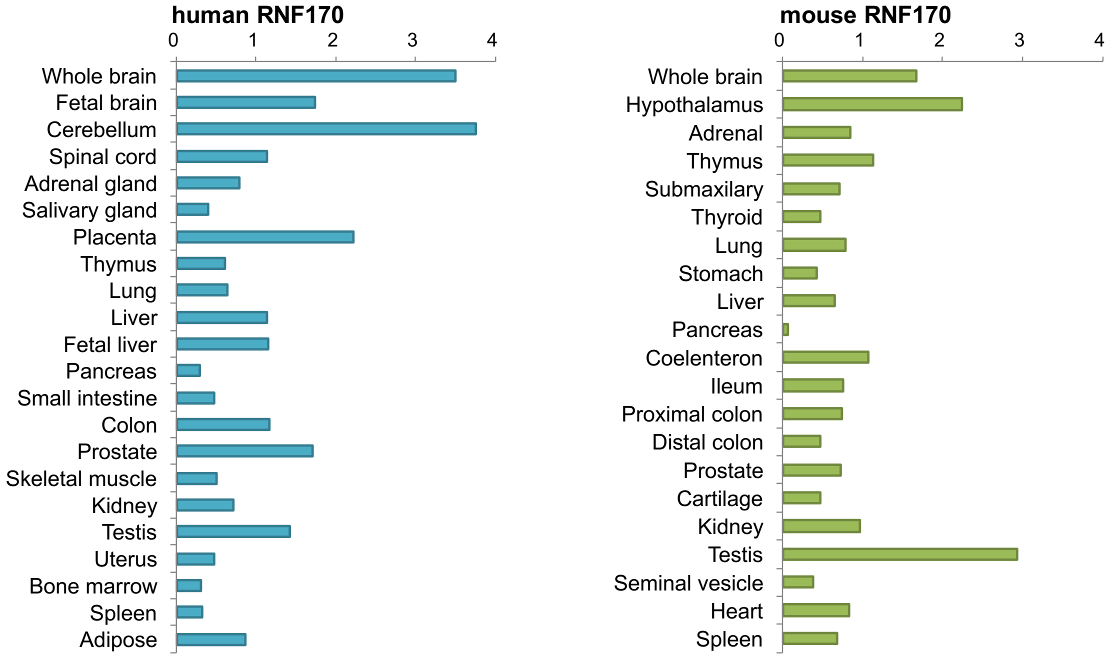
human RNF170
0
1
2
3
4
Whole brain
Fetal brain
Cerebellum
Spinal cord
Adrenal gland
Salivary gland
Placenta
Thymus
Lung
Liver
Fetal liver
Pancreas
Small intestine
Colon
Prostate
Skeletal muscle
Kidney
Testis
Uterus
Bone marrow
Spleen
Adipose
mouse RNF170
0
1
2
3
4
Whole brain
Hypothalamus
Adrenal
Thymus
Submaxilary
Thyroid
Lung
Stomach
Liver
Pancreas
Coelenteron
Ileum
Proximal colon
Distal colon
Prostate
Cartilage
Kidney
Testis
Seminal vesicle
Heart
Spleen

f

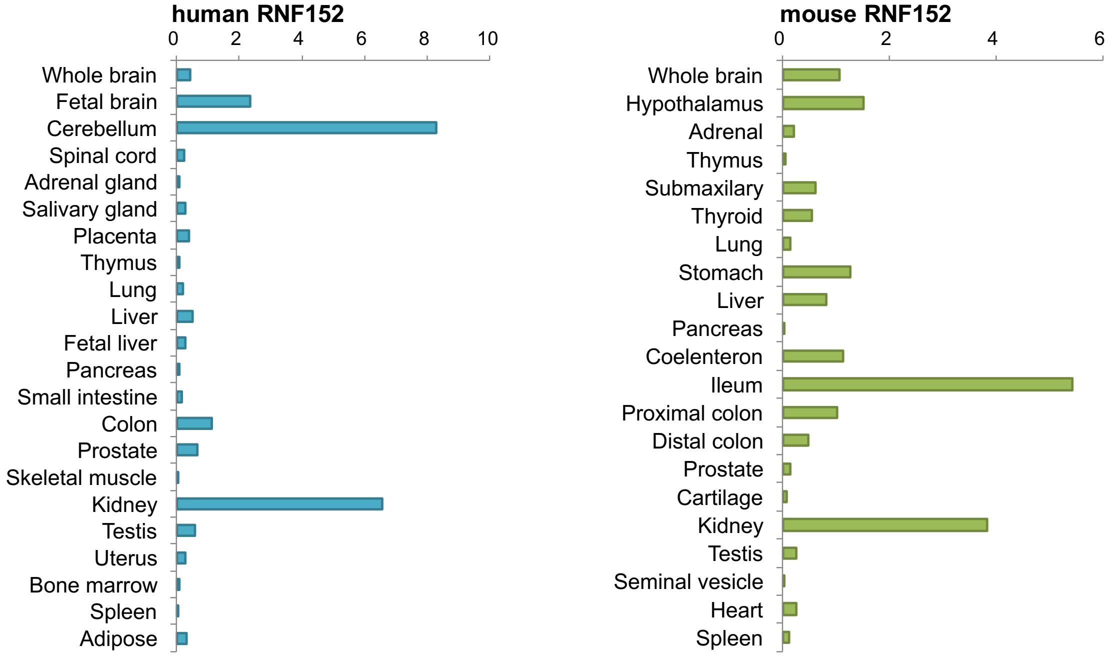
human RNF152
0
2
4
6
8
10
Whole brain
Fetal brain
Cerebellum
Spinal cord
Adrenal gland
Salivary gland
Placenta
Thymus
Lung
Liver
Fetal liver
Pancreas
Small intestine
Colon
Prostate
Skeletal muscle
Kidney
Testis
Uterus
Bone marrow
Spleen
Adipose
mouse RNF152
0
2
4
6
Whole brain
Hypothalamus
Adrenal
Thymus
Submaxilary
Thyroid
Lung
Stomach
Liver
Pancreas
Coelenteron
Ileum
Proximal colon
Distal colon
Prostate
Cartilage
Kidney
Testis
Seminal vesicle
Heart
Spleen

g

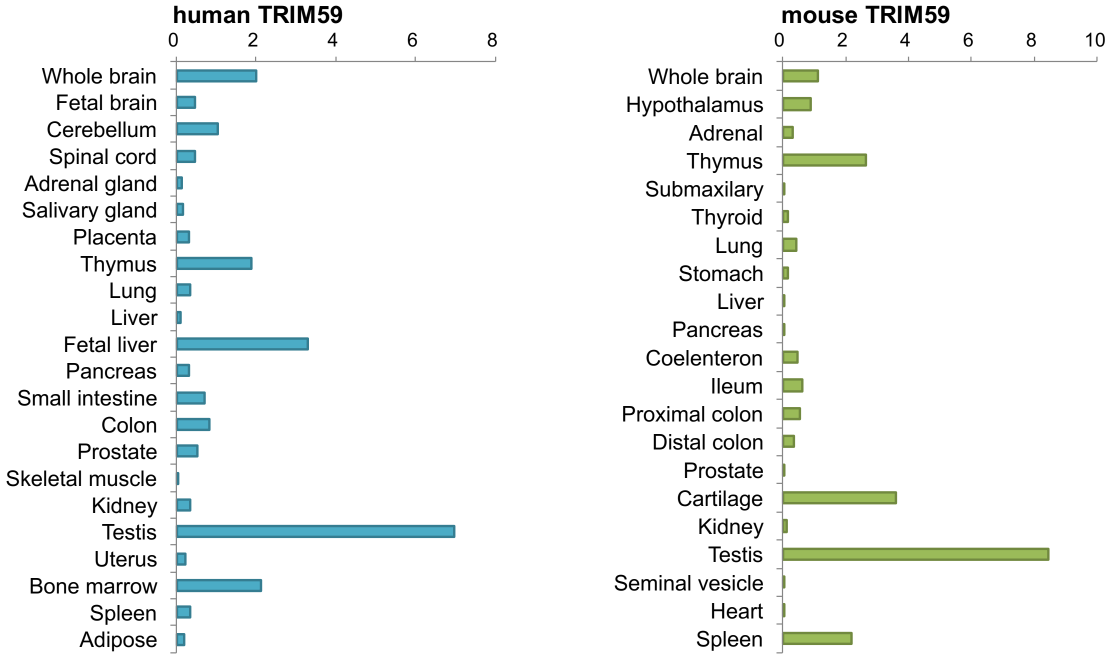
human TRIM59
0
2
4
6
8
Whole brain
Fetal brain
Cerebellum
Spinal cord
Adrenal gland
Salivary gland
Placenta
Thymus
Lung
Liver
Fetal liver
Pancreas
Small intestine
Colon
Prostate
Skeletal muscle
Kidney
Testis
Uterus
Bone marrow
Spleen
Adipose
mouse TRIM59
0
2
4
6
8
10
Whole brain
Hypothalamus
Adrenal
Thymus
Submaxilary
Thyroid
Lung
Stomach
Liver
Pancreas
Coelenteron
Ileum
Proximal colon
Distal colon
Prostate
Cartilage
Kidney
Testis
Seminal vesicle
Heart
Spleen

h

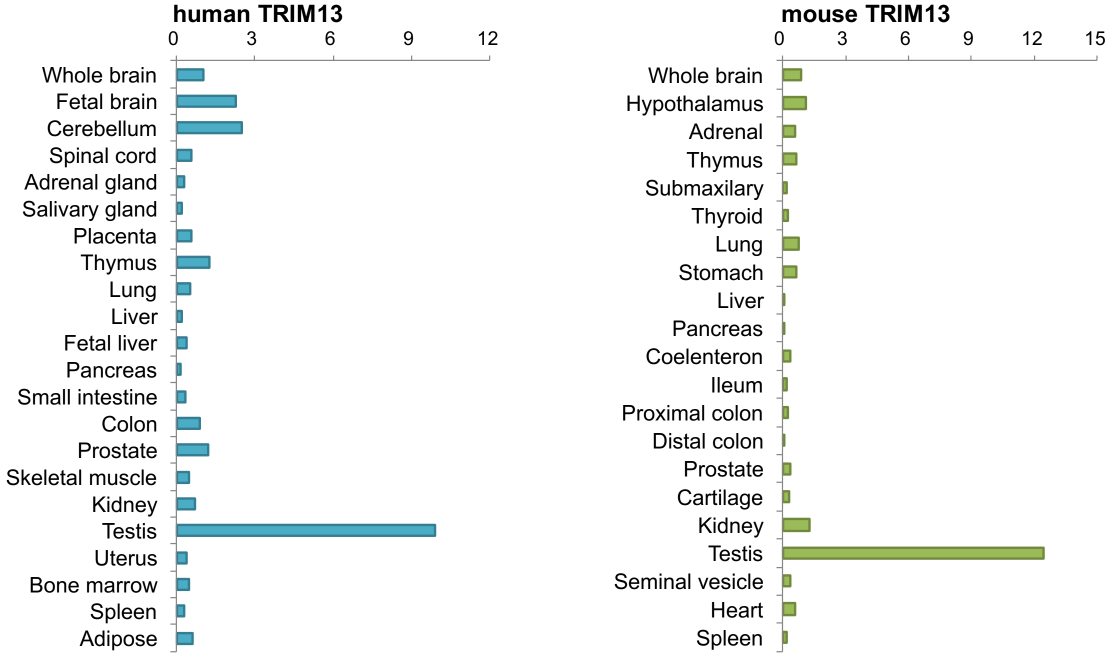
human TRIM13
0
3
6
9
12
Whole brain
Fetal brain
Cerebellum
Spinal cord
Adrenal gland
Salivary gland
Placenta
Thymus
Lung
Liver
Fetal liver
Pancreas
Small intestine
Colon
Prostate
Skeletal muscle
Kidney
Testis
Uterus
Bone marrow
Spleen
Adipose
mouse TRIM13
0
3
6
9
12
15
Whole brain
Hypothalamus
Adrenal
Thymus
Submaxilary
Thyroid
Lung
Stomach
Liver
Pancreas
Coelenteron
Ileum
Proximal colon
Distal colon
Prostate
Cartilage
Kidney
Testis
Seminal vesicle
Heart
Spleen

i

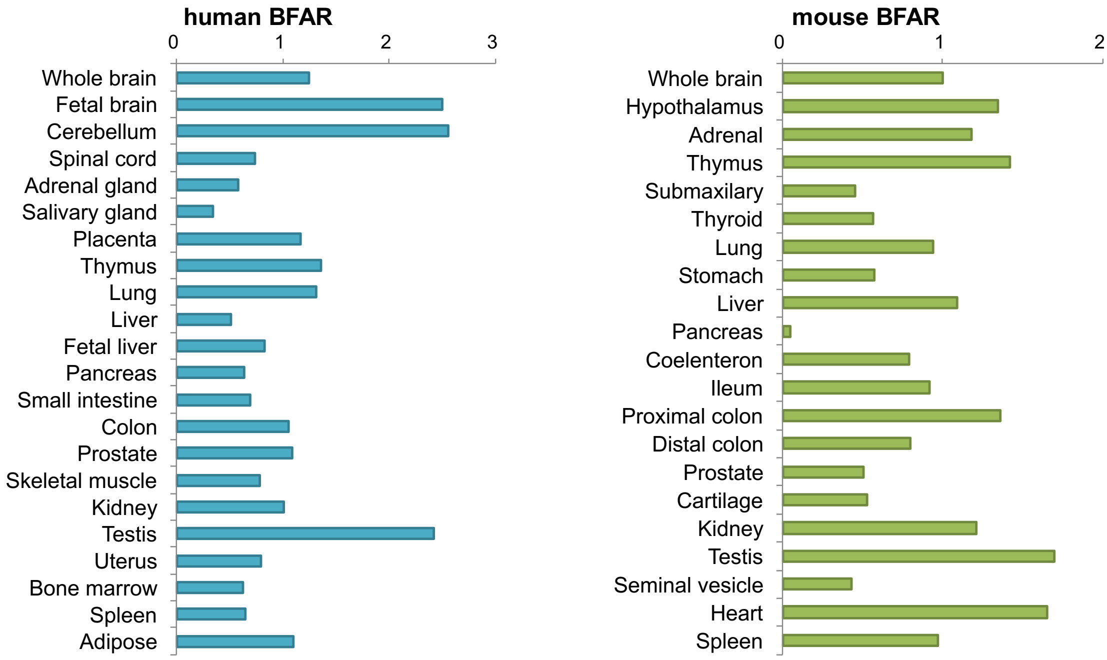
human BFAR
0
1
2
3
Whole brain
Fetal brain
Cerebellum
Spinal cord
Adrenal gland
Salivary gland
Placenta
Thymus
Lung
Liver
Fetal liver
Pancreas
Small intestine
Colon
Prostate
Skeletal muscle
Kidney
Testis
Uterus
Bone marrow
Spleen
Adipose
mouse BFAR
0
1
2
Whole brain
Hypothalamus
Adrenal
Thymus
Submaxilary
Thyroid
Lung
Stomach
Liver
Pancreas
Coelenteron
Ileum
Proximal colon
Distal colon
Prostate
Cartilage
Kidney
Testis
Seminal vesicle
Heart
Spleen

j

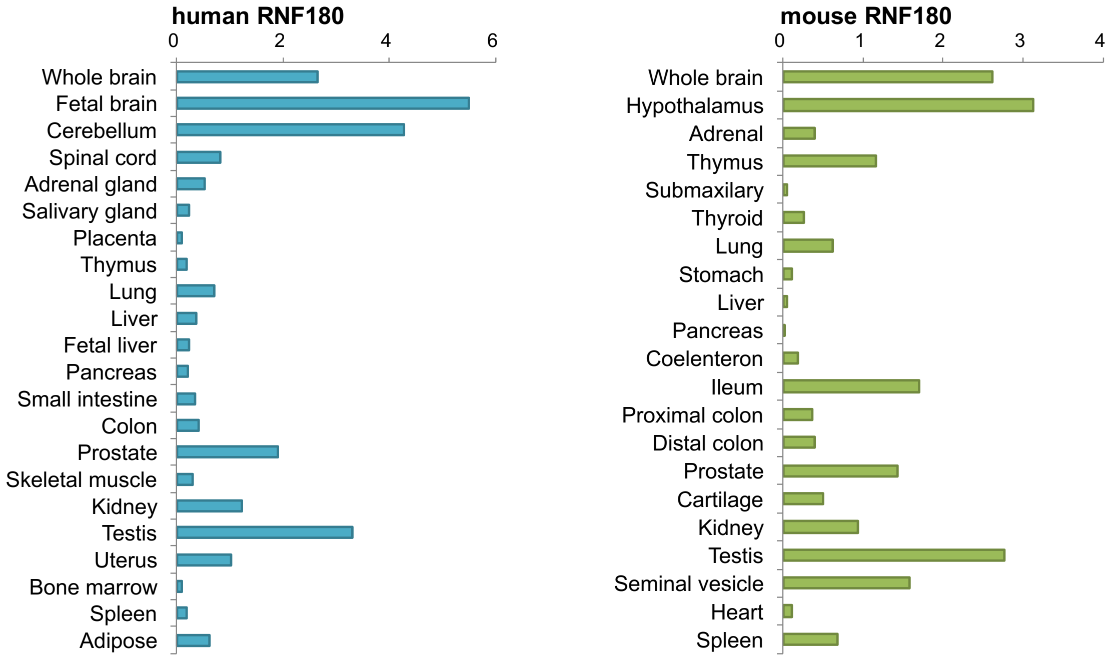
human RNF180
0
2
4
6
Whole brain
Fetal brain
Cerebellum
Spinal cord
Adrenal gland
Salivary gland
Placenta
Thymus
Lung
Liver
Fetal liver
Pancreas
Small intestine
Colon
Prostate
Skeletal muscle
Kidney
Testis
Uterus
Bone marrow
Spleen
Adipose
mouse RNF180
0
1
2
3
4
Whole brain
Hypothalamus
Adrenal
Thymus
Submaxilary
Thyroid
Lung
Stomach
Liver
Pancreas
Coelenteron
Ileum
Proximal colon
Distal colon
Prostate
Cartilage
Kidney
Testis
Seminal vesicle
Heart
Spleen

k

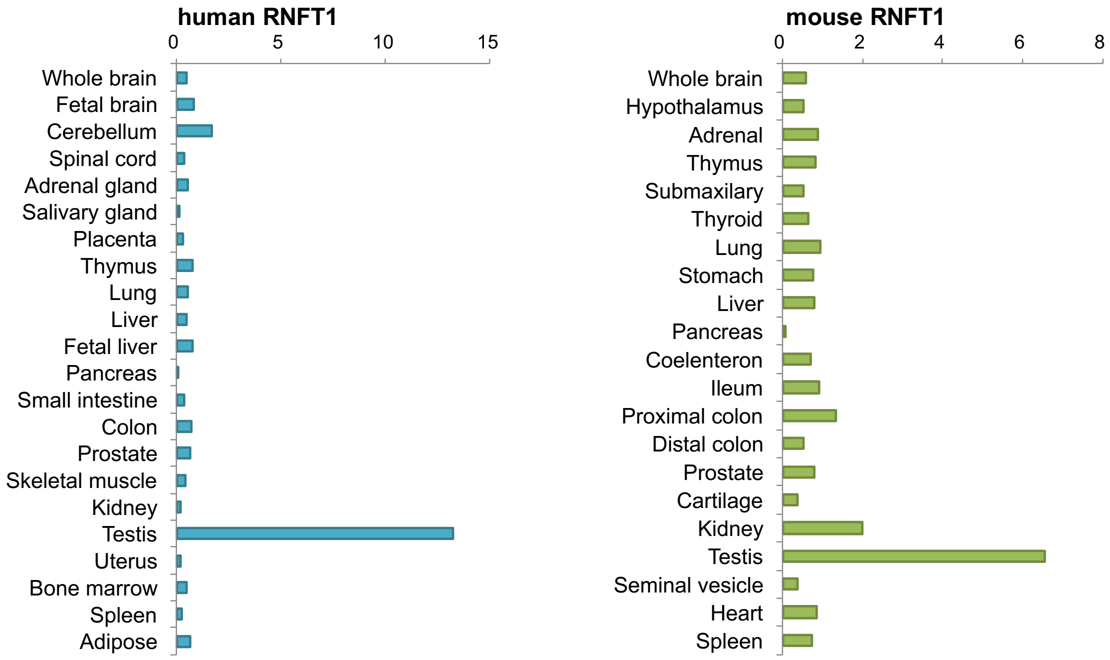

human RNFT1
0
5
10
15
Whole brain
Fetal brain
Cerebellum
Spinal cord
Adrenal gland
Salivary gland
Placenta
Thymus
Lung
Liver
Fetal liver
Pancreas
Small intestine
Colon
Prostate
Skeletal muscle
Kidney
Testis
Uterus
Bone marrow
Spleen
Adipose
mouse RNFT1
0
2
4
6
8
Whole brain
Hypothalamus
Adrenal
Thymus
Submaxilary
Thyroid
Lung
Stomach
Liver
Pancreas
Coelenteron
Ileum
Proximal colon
Distal colon
Prostate
Cartilage
Kidney
Testis
Seminal vesicle
Heart
Spleen

l

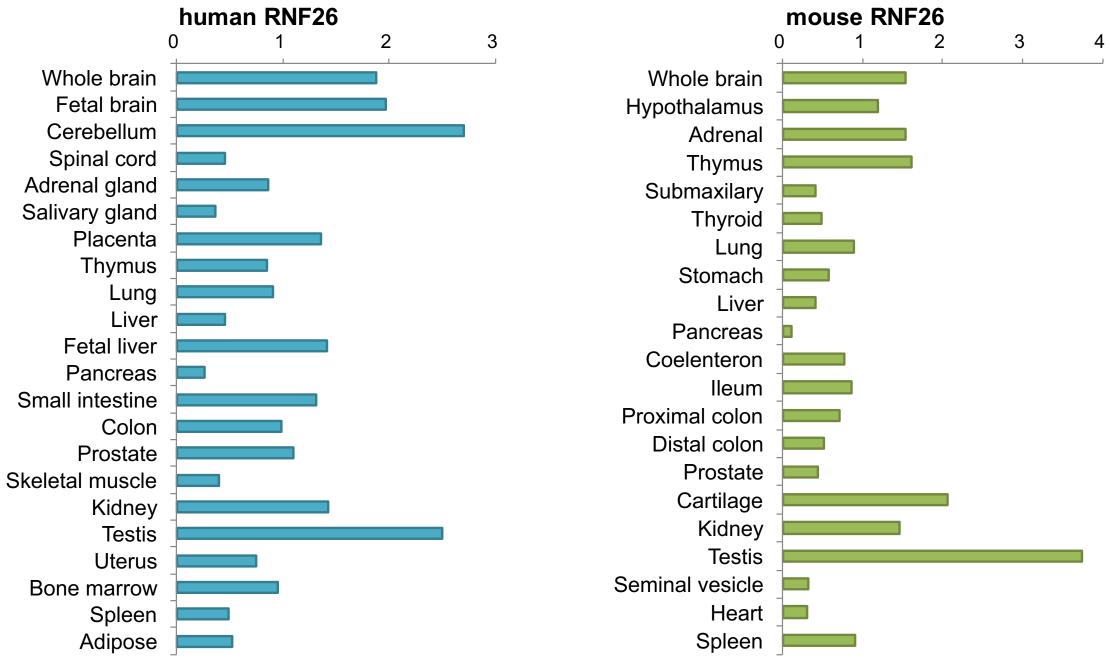

human RNF26
0
1
2
3
Whole brain
Fetal brain
Cerebellum
Spinal cord
Adrenal gland
Salivary gland
Placenta
Thymus
Lung
Liver
Fetal liver
Pancreas
Small intestine
Colon
Prostate
Skeletal muscle
Kidney
Testis
Uterus
Bone marrow
Spleen
Adipose
mouse RNF26
0
1
2
3
4
Whole brain
Hypothalamus
Adrenal
Thymus
Submaxilary
Thyroid
Lung
Stomach
Liver
Pancreas
Coelenteron
Ileum
Proximal colon
Distal colon
Prostate
Cartilage
Kidney
Testis
Seminal vesicle
Heart
Spleen

m

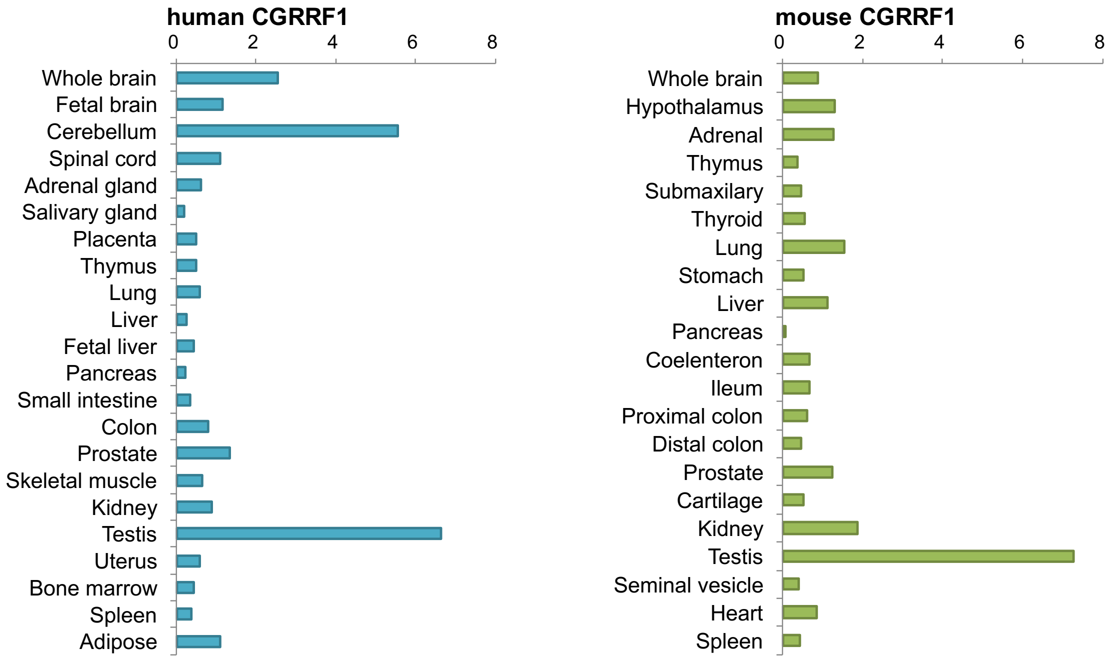
human CGRRF1
0 2 4 6 8
Whole brain
Fetal brain
Cerebellum
Spinal cord
Adrenal gland
Salivary gland
Placenta
Thymus
Lung
Liver
Fetal liver
Pancreas
Small intestine
Colon
Prostate
Skeletal muscle
Kidney
Testis
Uterus
Bone marrow
Spleen
Adipose
mouse CGRRF1
0 2 4 6 8
Whole brain
Hypothalamus
Adrenal
Thymus
Submaxilary
Thyroid
Lung
Stomach
Liver
Pancreas
Coelenteron
Ileum
Proximal colon
Distal colon
Prostate
Cartilage
Kidney
Testis
Seminal vesicle
Heart
Spleen

n

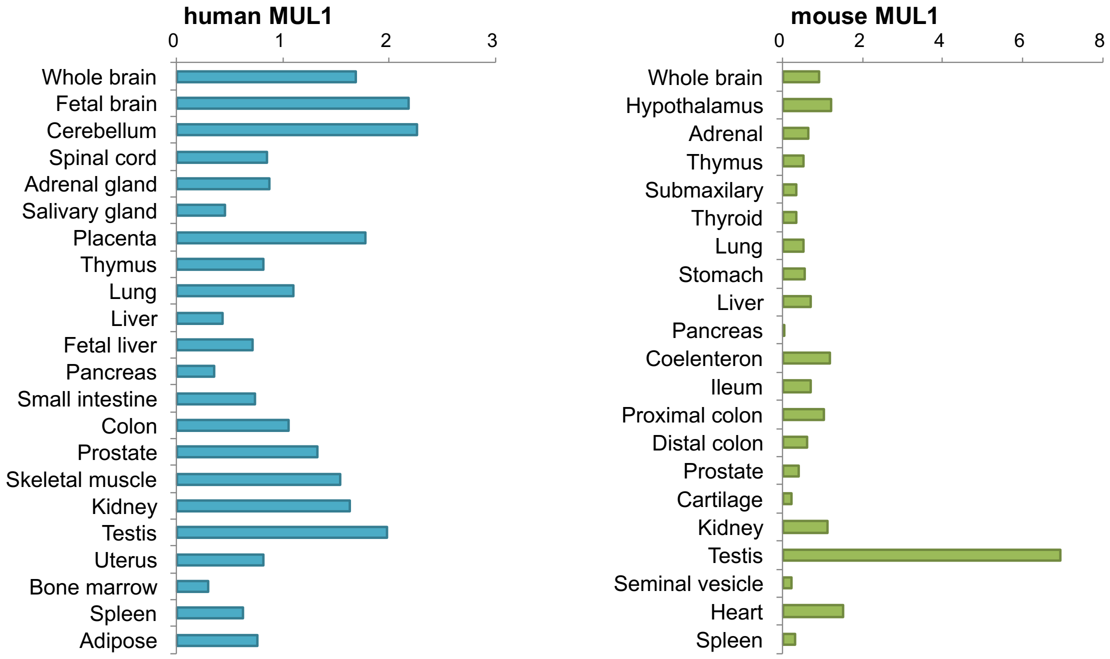
human MUL1
0 1 2 3
Whole brain
Fetal brain
Cerebellum
Spinal cord
Adrenal gland
Salivary gland
Placenta
Thymus
Lung
Liver
Fetal liver
Pancreas
Small intestine
Colon
Prostate
Skeletal muscle
Kidney
Testis
Uterus
Bone marrow
Spleen
Adipose
mouse MUL1
0 2 4 6 8
Whole brain
Hypothalamus
Adrenal
Thymus
Submaxilary
Thyroid
Lung
Stomach
Liver
Pancreas
Coelenteron
Ileum
Proximal colon
Distal colon
Prostate
Cartilage
Kidney
Testis
Seminal vesicle
Heart
Spleen

o

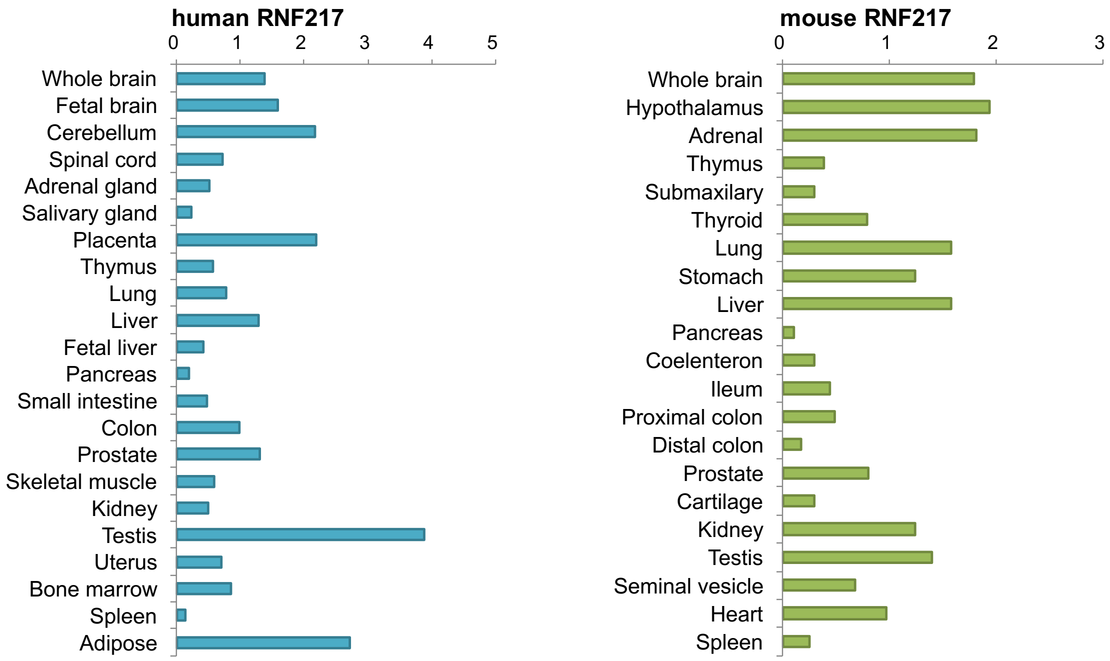

Supplementary Fig. S5. Tissue distribution of E3 ligases (C3HC4 type) in human and mouse tissues. Total RNAs from 23 human or 22 mouse tissues were reverse-transcribed and measured by TaqMan-based real-time PCR assay using the delta–delta Ct method. Data are normalised to the amount of 18S ribosomal RNA; results are expressed as the fold increase compared with cDNA pools from each tissue.

a

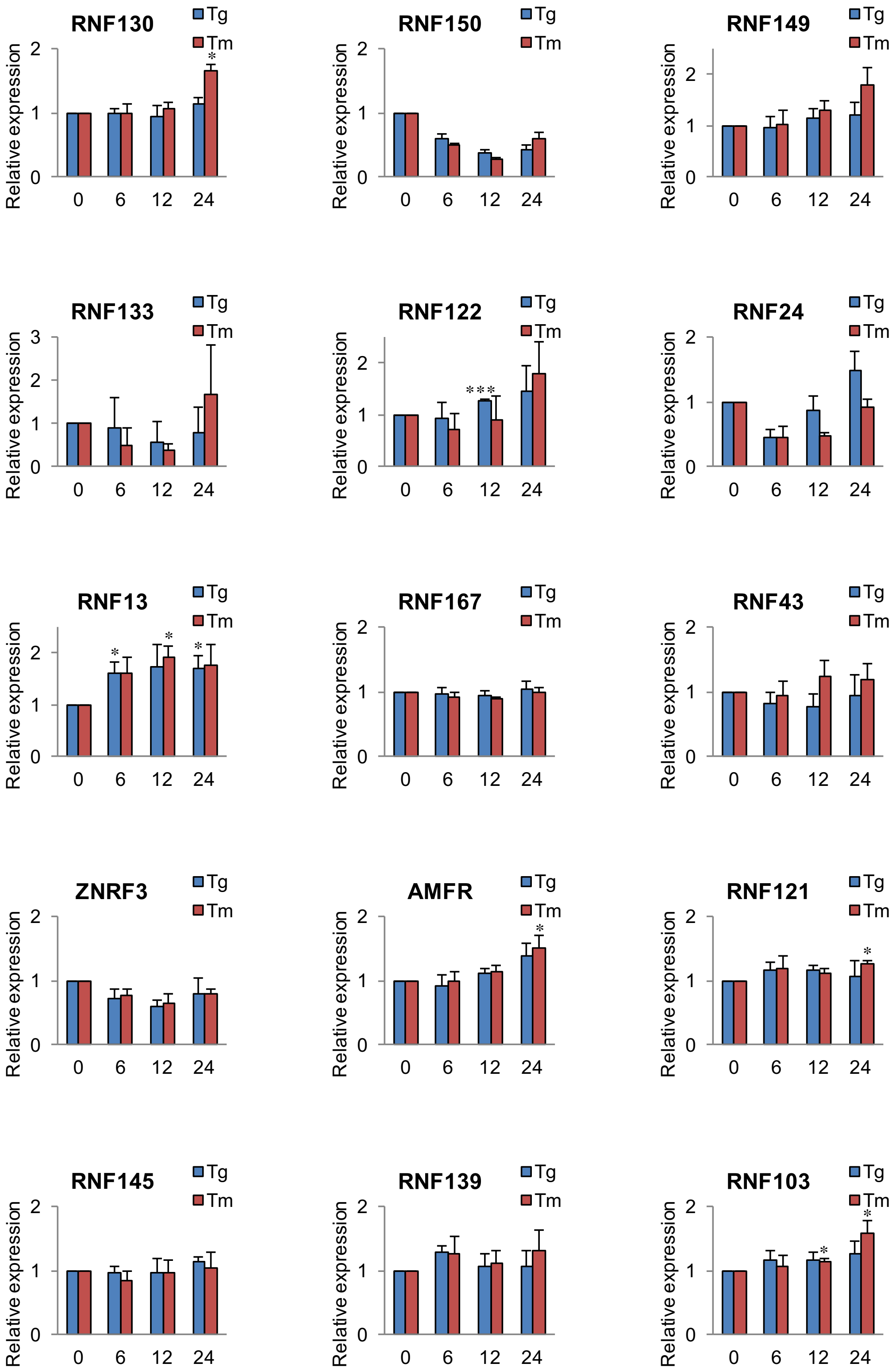
RNF130
RNF150
RNF149
RNF133
RNF122
RNF24
RNF13
RNF167
RNF43
ZNRF3
AMFR
RNF121
RNF145
RNF139
RNF103
Tg
Tm
Relative expression
0
6
12
24

b

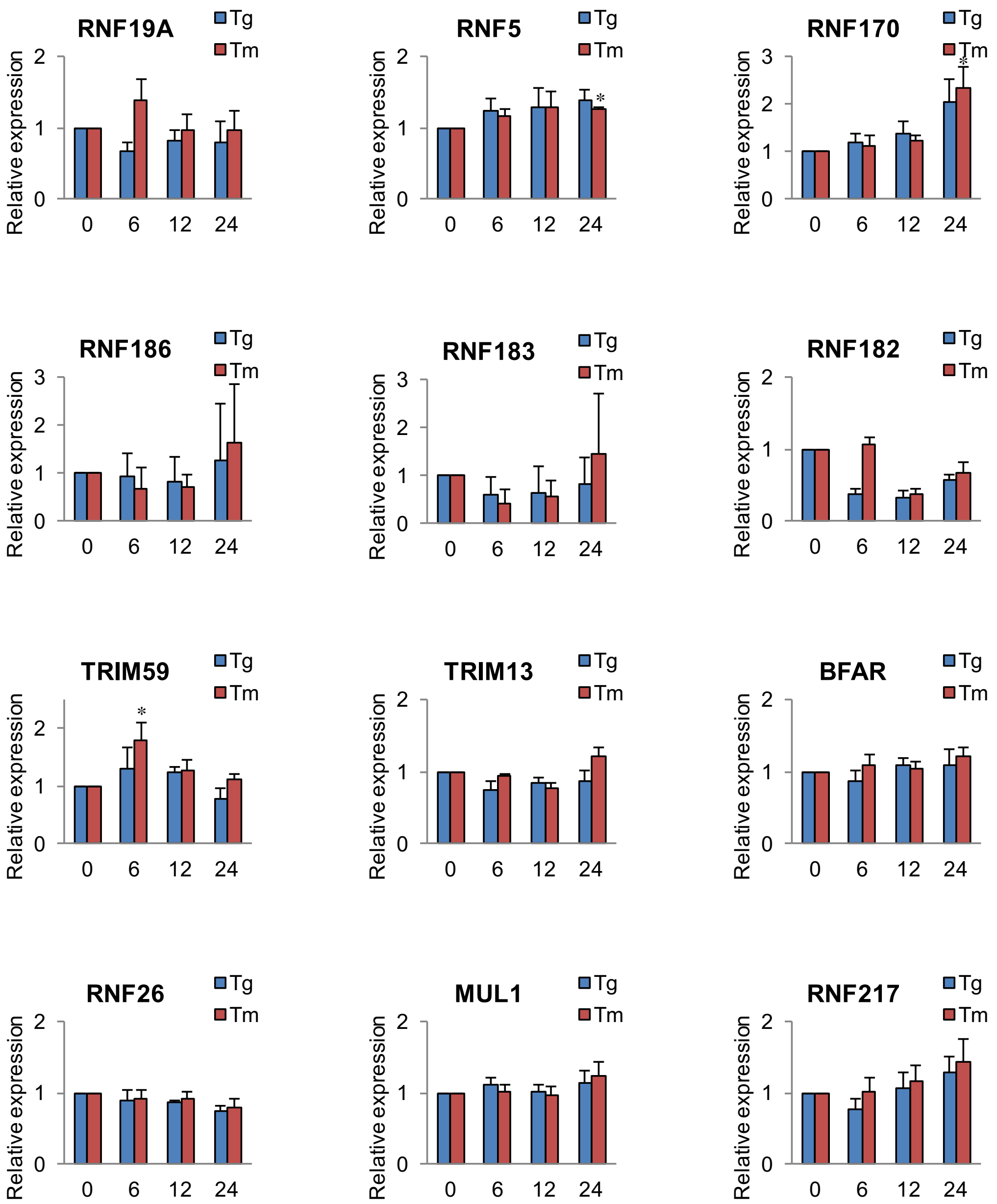

Supplementary Fig. S6. ER stress response of transmembrane E3 ligases. (**a**) C3H2C3-RING E3s. (**b**) C3HC4-RING E3s. Expression of E3 ligases induced by ER stressor. HeLa cells were treated with thapsigargin (Tg; 1 μM) and tunicamycin (Tm; 3 μg/ml) for the time periods as indicated. Total RNAs were reverse-transcribed and measured by TaqMan-based real-time PCR assay using the delta–delta Ct method. Data are normalized to the amount of GAPDH; results are expressed as the fold increase compared with control (mean ± SD; n = 4). Statistical analysis was performed with ANOVA followed by Bonferroni correction (vs. control; *p < 0.05, **p < 0.01, ***p < 0.001). Expression of RNF128, ZNRF4, RNF175, RNF152 and RNF180 was not detected in HeLa cells because their levels were below the detection limit.

a

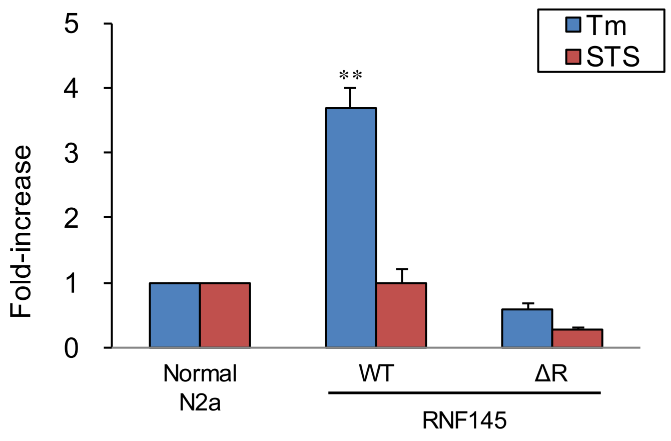

b

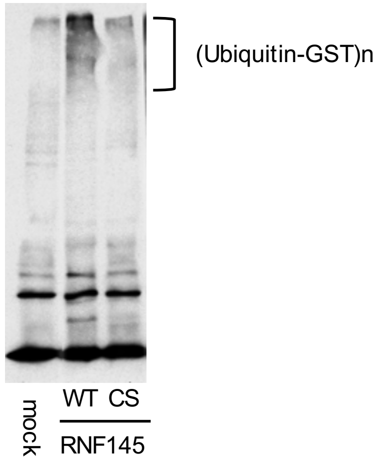

c

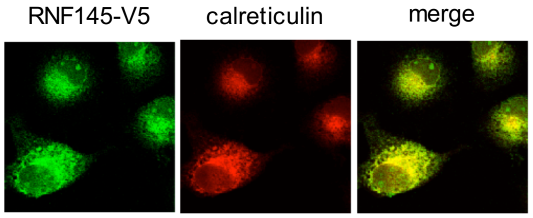

Supplementary Fig. S7. Characterisation of RNF145 for ERAD E3 ligase. (**a**) RNF145 protects against ER stress-induced cell death. Neuro-2a (N2a) cells (control) and those stably expressing WT or ΔRING (ΔR) mutant of RNF145 were treated with Tm (1 μg/ml) or staurosporine (STS; 0.2 μM) and incubated for 42 h. The cells were stained with crystal violet. The eluted dye at an optical density of 590 nm was measured. Cell viability was calculated as follows: OD for assay/OD for control well. The results are expressed as the fold increase compared with normal cells. The results are expressed as the means ± SD (three independent experiments in duplicate). Statistical analysis was performed with ANOVA followed by Bonferroni correction (vs. controls; $*p < 0.05$, $**p < 0.01$) (**b**) *In vitro* autoubiquitination assay. The RNF145 proteins produced by a transcription/translation system were mixed in the reaction buffer with E1 (rabbit), E2 (GST-UbcH5c) and GST-ubiquitin. The reaction mixture was immunoprecipitated with anti-V5 antibody and analysed by Western blotting using anti-GST antibody. CS mutants defective in E3 activity were constructed by replacement of conserved coordinating Cys with Ser residues in the RING (**c**) Subcellular localization of RNF145. COS-1 cells transiently transfected with RNF145-V5. The cells were subjected to immunofluorescence staining with anti-V5 and calreticulin antibodies.

Supplementary Table S1a Tissue distribution of transmembrane E3 ligases (human C3H2C3 type).

	Whole brain	Fetal brain	Cerebe-llum	Spinal cord	Adrenal gland	Salivary gland	Placen-ta	Thymus	Lung	Liver	Fetal liver	Pancre-as	Small intestine	Colon	Prostate	Skeletal muscle	Kidney	Testis	Uterus	Bone marrow	Spleen	Adipose
RNF130	3.89	0.48	2.51	1.87	0.57	0.52	0.84	0.24	0.64	0.99	0.65	0.35	0.56	0.59	0.55	0.42	1.70	0.88	0.54	3.11	0.45	1.55
RNF150	1.91	5.51	4.27	0.55	1.75	1.70	0.13	0.38	0.24	0.05	0.11	0.06	0.49	0.87	1.20	0.91	0.99	0.74	1.23	0.03	0.40	0.91
RNF149	1.40	1.09	1.58	0.64	0.68	0.42	2.04	1.57	2.12	0.61	0.83	0.28	0.55	1.08	1.38	0.26	1.11	2.92	0.66	1.25	1.36	0.99
RNF133	0.33	0.09	9.10	0.11	0.08	0.13	0.07	0.07	0.19	0.09	0.10	0.10	0.14	0.23	0.52	0.01	0.30	14.23	0.26	0.02	0.01	0.20
RNF128	0.88	0.24	0.40	1.23	1.29	0.85	1.04	0.19	0.45	4.73	3.30	0.51	1.74	2.77	0.42	0.67	1.77	0.17	0.00	0.06	0.00	0.02
RNF122	0.31	2.60	4.57	0.57	1.43	0.40	0.31	0.79	2.41	0.21	0.62	0.21	0.58	0.59	0.87	2.32	0.60	1.37	0.80	0.36	0.84	0.95
RNF24	4.30	7.70	2.42	3.49	0.35	0.12	0.30	0.58	0.52	0.01	0.26	0.01	0.12	0.16	0.72	0.12	0.13	0.62	0.26	1.39	0.15	2.69
RNF13	4.24	0.55	3.58	2.61	0.39	0.44	1.22	0.69	0.71	0.71	0.64	0.35	0.38	1.01	1.85	0.49	1.07	2.79	0.31	0.47	0.32	1.30
RNF167	1.89	1.76	2.10	0.71	0.82	0.48	1.33	1.15	0.94	0.70	0.89	0.23	0.85	0.98	1.29	0.77	1.09	3.16	0.65	0.79	0.58	0.68
ZNRF4	0.01	0.14	0.01	0.00	0.00	0.00	0.00	0.00	0.00	0.00	0.00	0.00	0.00	0.00	0.00	0.00	0.00	24.60	0.00	0.00	0.00	0.00
RNF43	0.70	0.13	4.76	0.17	1.94	0.80	1.60	0.64	0.65	1.13	0.72	0.32	3.05	2.57	2.68	0.02	0.89	1.71	0.18	0.04	0.04	0.02
ZNRF3	2.39	2.34	4.15	0.80	1.36	0.62	1.12	0.25	0.71	0.76	0.79	0.25	0.79	0.53	1.89	0.24	2.78	2.64	0.81	0.11	0.12	0.51
SYVN1	1.17	1.27	1.79	0.57	1.08	0.74	2.00	0.82	1.01	0.96	1.21	0.68	0.73	0.90	1.46	0.51	0.82	2.14	0.50	0.46	0.76	0.76
AMFR	1.77	1.92	2.07	0.80	1.09	0.25	2.24	0.40	0.66	0.72	0.65	0.23	0.49	0.63	0.93	1.56	1.67	4.25	0.62	0.30	0.39	0.59
RNF175	9.69	8.05	8.07	0.65	0.10	0.07	0.01	0.01	0.02	0.00	0.02	1.83	0.03	0.04	0.31	0.00	0.00	1.44	0.02	0.07	0.01	0.20
RNF121	1.76	3.00	2.93	0.62	0.74	0.47	1.18	0.96	0.80	0.41	0.79	0.22	0.91	0.75	1.32	0.81	1.94	2.94	0.63	0.52	0.38	0.48
RNF145	2.23	4.86	3.44	1.09	0.90	0.28	1.79	1.25	1.47	0.13	0.82	0.10	0.30	1.03	1.31	0.15	0.26	1.45	0.50	0.79	0.57	0.79
RNF139	1.36	2.11	2.18	0.50	0.84	0.27	1.33	0.88	0.77	0.63	0.54	0.26	0.51	0.73	0.98	0.78	0.84	7.08	0.69	0.43	0.37	0.77
RNF103	2.14	2.33	5.93	0.69	0.48	0.19	1.19	0.42	0.60	0.85	0.50	0.36	0.59	1.21	1.47	0.50	0.68	2.31	0.77	0.34	0.23	0.53

Supplementary Table S1b Tissue distribution of transmembrane E3 ligases (human C3HC4 type).

	Whole brain	Fetal brain	Cerebe-llum	Spinal cord	Adrenal gland	Salivary gland	Placen-ta	Thymus	Lung	Liver	Fetal liver	Pancre-as	Small intestine	Colon	Prostate	Skeletal muscle	Kidney	Testis	Uterus	Bone marrow	Spleen	Adipose
RNF19A	2.14	1.97	5.13	1.33	0.15	0.08	0.39	0.94	0.48	0.33	1.37	0.18	0.34	0.79	0.70	0.39	0.59	6.00	0.39	0.64	0.21	0.73
RNF19B	1.27	2.86	3.15	0.61	0.39	0.13	0.72	0.38	0.93	0.11	0.19	0.11	0.53	0.90	0.54	0.28	0.79	9.76	0.39	0.25	0.37	0.38
RNF5	1.51	2.56	2.01	0.47	0.76	0.33	0.53	0.71	0.59	0.72	0.94	0.14	0.65	0.76	1.23	0.87	1.79	1.30	0.40	0.51	0.34	0.39
RNF185	1.47	1.94	2.25	0.67	0.66	0.79	1.46	0.81	1.15	0.66	0.58	0.21	0.71	1.06	3.09	0.58	1.60	2.10	0.89	0.24	0.50	0.73
RNF170	3.51	1.73	3.75	1.14	0.79	0.40	2.22	0.60	0.64	1.13	1.15	0.30	0.47	1.17	1.71	0.51	0.72	1.43	0.47	0.31	0.33	0.87
RNF186	1.40	0.01	5.29	0.03	0.00	0.01	0.05	0.01	0.03	0.12	0.02	5.44	4.99	4.37	0.02	0.00	4.45	0.23	0.01	0.17	0.00	0.01
RNF152	0.45	2.34	8.28	0.25	0.08	0.27	0.39	0.08	0.21	0.51	0.28	0.11	0.16	1.13	0.68	0.06	6.57	0.59	0.27	0.09	0.01	0.33
RNF183	0.11	0.33	0.58	0.15	0.04	0.17	0.14	0.10	0.46	0.06	0.08	0.08	0.15	0.26	0.68	0.01	8.68	8.96	0.46	0.02	0.02	0.02
RNF182	2.85	9.35	10.06	1.04	0.02	0.04	0.14	0.31	0.59	0.00	0.56	0.03	0.10	0.02	0.04	0.02	0.40	2.08	0.03	1.16	0.00	0.14
TRIM59	2.00	0.45	1.04	0.46	0.13	0.17	0.32	1.87	0.35	0.09	3.30	0.32	0.69	0.84	0.51	0.02	0.33	6.95	0.23	2.13	0.35	0.20
TRIM13	1.05	2.28	2.52	0.55	0.29	0.20	0.58	1.27	0.54	0.20	0.40	0.15	0.32	0.89	1.21	0.47	0.69	9.88	0.39	0.48	0.30	0.61
BFAR	1.25	2.50	2.56	0.73	0.58	0.35	1.17	1.36	1.31	0.52	0.83	0.64	0.70	1.05	1.09	0.78	1.01	2.42	0.80	0.62	0.65	1.10
RNF180	2.66	5.49	4.28	0.83	0.53	0.23	0.09	0.19	0.71	0.36	0.24	0.21	0.36	0.42	1.90	0.30	1.23	3.30	1.03	0.11	0.18	0.62
RNFT1	0.46	0.81	1.69	0.38	0.53	0.16	0.31	0.78	0.54	0.48	0.76	0.10	0.35	0.71	0.63	0.43	0.20	13.20	0.20	0.46	0.23	0.63
RNF26	1.88	1.97	2.70	0.46	0.86	0.36	1.36	0.85	0.91	0.46	1.42	0.27	1.31	0.98	1.10	0.40	1.43	2.50	0.75	0.95	0.49	0.52
CGRRF1	2.53	1.15	5.54	1.10	0.62	0.21	0.50	0.51	0.59	0.25	0.44	0.22	0.35	0.80	1.35	0.64	0.88	6.62	0.60	0.42	0.37	1.10
MUL1	1.69	2.18	2.26	0.86	0.87	0.46	1.78	0.82	1.10	0.44	0.71	0.35	0.74	1.06	1.33	1.54	1.63	1.98	0.81	0.30	0.63	0.76
RNF217	1.38	1.60	2.18	0.72	0.51	0.23	2.19	0.58	0.79	1.29	0.43	0.20	0.48	0.99	1.30	0.59	0.50	3.88	0.70	0.85	0.13	2.72

Supplementary Table S1c Tissue distribution of transmembrane E3 ligases (mouse C3H2C3 type).

	Whole brain	Hypotha-lamus	Adrenal	Thymus	Sub-maxilary	Thyroid	Lung	Stomach	Liver	Pancre-as	Coelen-teron	Ileum	Proximal colon	Distal colon	Prostate	Cartilage	Kidney	Testis	Seminal vesicle	Heart	Spleen
RNF130	1.72	1.66	1.40	1.02	0.48	1.12	1.13	0.76	0.79	0.05	0.24	0.36	0.75	0.58	1.83	1.44	1.76	0.87	0.32	0.78	1.21
RNF150	4.67	2.54	1.73	0.53	0.04	0.36	1.58	0.16	0.03	0.01	0.06	0.13	0.63	0.14	0.09	0.32	0.13	0.18	0.08	1.31	1.06
RNF149	0.75	0.86	0.90	0.08	0.83	0.94	0.22	0.69	0.94	1.09	1.08	0.92	0.71	1.06	0.84	2.11	1.28	1.54	1.54	0.79	2.20
RNF133	0.17	0.15	0.01	0.01	0.17	0.08	0.12	0.04	0.02	0.01	0.29	0.07	0.10	0.11	0.13	0.02	0.03	25.05	0.03	0.01	0.02
RNF128	0.27	0.59	0.76	0.16	0.60	0.71	0.13	1.67	0.93	0.02	3.71	3.93	1.83	0.88	0.62	0.03	2.36	0.19	0.73	1.17	0.02
RNF122	0.90	1.01	1.24	1.34	0.95	0.65	2.64	0.41	0.08	0.06	0.57	0.72	0.48	0.32	0.34	0.75	0.94	0.29	0.31	0.97	3.56
RNF24	1.78	1.83	0.52	0.26	0.10	0.55	0.33	0.11	0.16	0.01	0.22	0.39	0.25	0.31	0.08	0.24	3.12	9.68	0.08	0.19	0.12
RNF13	1.37	1.44	1.34	0.86	0.56	0.85	1.46	0.58	1.35	0.06	0.73	0.55	0.53	0.33	0.70	0.43	2.78	0.84	0.29	1.30	0.81
RNF167	1.03	1.17	1.32	2.77	0.46	0.71	1.28	0.47	0.47	0.05	1.14	0.81	0.72	0.36	0.46	1.17	1.38	3.49	0.48	0.70	1.42
ZNRF4	0.00	0.00	0.00	0.00	0.00	0.01	0.00	0.00	0.00	0.00	0.25	0.02	0.00	0.00	0.02	0.00	0.00	30.68	0.00	0.00	0.00
RNF43	0.34	0.17	0.71	0.73	2.63	0.88	1.53	1.30	2.15	0.05	2.60	2.27	2.02	1.95	1.23	0.06	0.70	1.16	1.55	0.00	0.20
ZNRF3	1.28	0.92	2.16	1.83	0.69	0.82	1.48	0.71	0.53	0.10	0.52	0.55	0.55	0.47	0.77	0.23	1.26	4.63	0.73	0.48	0.52
SYVN1	1.00	1.18	1.34	1.07	0.84	0.37	0.80	0.56	0.83	0.15	0.92	1.16	0.84	0.96	1.37	0.82	0.64	2.80	0.92	0.45	1.82
AMFR	1.10	1.12	1.75	0.60	0.51	0.64	0.90	0.45	1.82	0.05	0.54	0.70	0.63	0.47	0.58	0.58	1.81	3.30	0.30	1.09	0.60
RNF121	1.47	2.01	1.00	1.00	0.59	0.41	0.85	0.76	0.55	0.03	2.09	0.94	1.04	0.82	0.55	0.38	1.06	3.36	0.33	0.65	0.62
RNF145	1.38	1.69	4.26	0.04	0.53	0.46	1.88	0.28	0.31	0.84	0.68	0.69	0.66	2.04	0.37	1.06	1.65	0.21	1.93	0.23	0.25
RNF139	0.45	0.49	0.48	0.77	0.17	0.29	0.60	0.30	0.39	0.04	0.59	0.34	0.36	0.24	0.34	0.78	0.74	14.06	0.17	0.63	0.63
RNF103	1.25	1.33	0.90	0.64	0.91	0.44	0.80	0.65	1.48	0.04	0.97	1.44	1.70	1.16	1.54	0.31	1.15	0.80	0.55	0.56	0.43

e

Supplementary Table S1d Tissue distribution of transmembrane E3 ligases (mouse C3HC4 type).

	Whole brain	Hypotha-lamus	Adrenal	Thymus	Sub-maxilary	Thyroid	Lung	Stomach	Liver	Pancre-as	Coelen-teron	Ileum	Proximal colon	Distal colon	Prostate	Cartilage	Kidney	Testis	Seminal vesicle	Heart	Spleen
RNF19A	0.43	0.41	0.48	0.72	0.09	0.30	0.78	0.20	0.26	0.01	0.58	0.49	0.27	0.17	0.15	0.47	0.62	17.33	0.11	0.30	0.41
RNF19B	0.36	0.37	0.26	0.38	0.16	0.11	0.45	0.18	0.27	0.02	0.67	0.69	0.45	0.37	0.14	0.33	0.30	16.40	0.07	0.20	0.63
RNF5	1.08	1.59	1.22	1.07	0.76	1.07	0.59	0.48	1.25	0.12	1.37	1.44	1.05	0.89	0.59	0.56	2.27	1.28	0.51	0.66	0.59
RNF185	0.88	1.13	1.26	0.69	0.77	0.73	0.98	0.62	0.70	0.09	0.97	1.05	0.73	0.55	1.16	0.41	1.85	5.22	0.82	0.67	0.64
RNF170	1.67	2.25	0.84	1.12	0.71	0.47	0.79	0.42	0.64	0.07	1.07	0.75	0.74	0.48	0.72	0.46	0.96	2.93	0.38	0.83	0.68
RNF186	0.00	0.01	0.00	0.06	0.00	0.05	1.59	0.44	0.45	0.02	3.84	2.66	8.66	4.33	2.45	0.04	2.20	0.01	0.03	0.01	0.08
RNF152	1.06	1.51	0.20	0.07	0.61	0.56	0.14	1.28	0.83	0.01	1.13	5.43	1.01	0.48	0.15	0.08	3.83	0.26	0.04	0.26	0.12
RNF183	0.03	0.22	0.44	0.81	0.00	2.44	0.13	0.02	0.00	0.01	0.07	0.05	0.07	0.30	0.01	0.00	21.98	1.63	0.01	0.03	0.01
RNF182	4.76	4.31	0.27	0.05	0.07	0.27	0.02	0.04	0.01	0.03	0.02	0.03	0.03	0.02	0.31	0.31	0.13	1.96	0.13	0.04	0.07
TRIM59	1.12	0.88	0.31	2.66	0.06	0.16	0.44	0.15	0.01	0.02	0.48	0.63	0.54	0.37	0.07	3.61	0.12	8.43	0.06	0.07	2.18
TRIM13	0.88	1.13	0.62	0.66	0.21	0.25	0.75	0.64	0.07	0.05	0.36	0.21	0.27	0.11	0.37	0.31	1.29	12.41	0.38	0.60	0.18
BFAR	1.00	1.34	1.18	1.42	0.46	0.57	0.94	0.57	1.09	0.05	0.79	0.92	1.36	0.80	0.50	0.53	1.21	1.70	0.43	1.65	0.97
RNF180	2.61	3.12	0.40	1.16	0.06	0.27	0.63	0.12	0.06	0.01	0.19	1.71	0.37	0.40	1.43	0.50	0.94	2.76	1.58	0.12	0.68
RNFT1	0.58	0.53	0.89	0.82	0.52	0.63	0.93	0.77	0.78	0.07	0.69	0.93	1.34	0.53	0.79	0.38	1.99	6.55	0.37	0.85	0.74
RNF26	1.53	1.19	1.53	1.61	0.42	0.49	0.89	0.58	0.41	0.11	0.77	0.87	0.70	0.52	0.44	2.07	1.46	3.74	0.33	0.31	0.91
CGRRF1	0.88	1.29	1.27	0.36	0.46	0.57	1.54	0.51	1.14	0.08	0.67	0.66	0.62	0.47	1.23	0.52	1.89	7.29	0.41	0.86	0.43
MUL1	0.92	1.22	0.64	0.53	0.35	0.36	0.53	0.56	0.71	0.06	1.18	0.72	1.04	0.62	0.41	0.23	1.14	6.95	0.23	1.51	0.30
RNF217	1.79	1.94	1.82	0.39	0.30	0.79	1.58	1.24	1.58	0.11	0.30	0.45	0.48	0.18	0.80	0.30	1.24	1.39	0.68	0.97	0.25

Supplementary Table S2 Primer sets used for the cloning of WT, ΔR and CS constructs.

gene	primer	WT	ΔR	CS
RNFT1	Forward	5'-CACCATGCCGCTGTTCTTGCTGTC-3'	5'-AGAACTGTGATTTCAGACCAT-3'	5'-GATATTTCTTCAATATCTCAAGCTGAATTTCAGAAGCC-3'
	Reverse	5'-ATATATTTGAAGGTGTGATGAA-3'	5'-AATATCATCCACATCTGAACAC-3'	5'-GGCTTCTGAAATTCAGCTTGAGATATTGAAGAAATATC-3'
RNF185	Forward	5'-CACCATGGCAAGCAAGGGGC-3'	5'-AAAGCTGGCATCAGCCGAGA-3'	5'-GGACAGCACTTTCGAGTCCAACATCTGCTTGG-3'
	Reverse	5'-GGCAATCAGGAGCCAGAACA-3'	5'-CTCGAAAGTGCTGTCCTGCC-3'	5'-CCAAGCAGATGTTGGACTCGAAAGTGCTGTCC-3'
CGRRF1	Forward	5'-CACCATGGCTGCGGTGTTTCTGGT-3'	5'-AGGCAGTTTGTTCAGGAATC-3'	5'-GCAAGGACTCTGTTGTTTCCCAGAATGGG-3'
	Reverse	5'-AAGAGTCTTCGGTTTGTCTTTA-3'	5'-GTCCTTGCTGTTCTCTTCCG-3'	5'-CCCATTCTGGGAAACAACAGAGTCCTTGC-3'
RNF19B	Forward	5'-CACCATGGGCTCCGAGAAGGACT-3'	5'-CACCATGAAAGAGATCTCAGACTTGC-3'	
	Reverse	5'-TACTCTGGCTTCTCCACCTT-3'	5'-TACTCTGGCTTCTCCACCTTC-3'	

Supplementary Table S3a Sets of primers and probes for real-time PCR (C3H2C3 type).

Gene Name	AB Assay ID	IDT Assay ID
RNF130	Hs00218335_m1	Mm.PT.58.9219868
RNF150	Hs00324941_m1	Mm.PT.58.10273312
RNF149	Hs00411860_m1	Mm.PT.58.11306683
RNF133	Hs00373669_s1	Mm.PT.58.43776563.g
RNF128	Hs00226053_m1	Mm.PT.56a.8462982
RNF122	Hs00227141_m1	Mm.PT.58.8505793
RNF24	Hs00200579_m1	Mm.PT.58.9635653
RNF13	Hs00961507_m1	Mm.PT.58.11306683
RNF167	Hs00248913_m1	Mm.PT.58.32670381
ZNRF4	Hs00741333_s1	Mm.PT.58.32478833.g
RNF43	Hs00214886_m1	Mm.PT.56a.31556976
ZNRF3	Hs00293334_m1	Mm.PT.56a.5645486
SYVN1	Hs00381211_m1	Mm.PT.58.14132695
AMFR	Hs01031688_m1	Mm.PT.58.9856954
RNF175	Hs00922707_m1	
RNF121	Hs00217843_m1	Mm.PT.58.6378114
RNF145	Hs01099640_m1	Mm.PT.58.10110397
RNF139	Hs00183680_m1	Mm.PT.58.45812642
RNF103	Hs01084476_m1	Mm.PT.58.9763784

Supplementary Table S3b Sets of primers and probes for real-time PCR (C3HC4 type).

Gene Name	AB Assay ID	IDT Assay ID
RNF19A	Hs00209954_m1	Mm.PT.58.28600163
RNF19B	Hs00415359_m1	Mm.PT.58.28736280
RNF5	Hs00359834_g1	Mm.PT.58.14209781
RNF185	Hs01565008_m1	Mm.PT.56a.11642500
RNF170	Hs00229577_m1	Mm.PT.56a.13272002
RNF186	Hs00535508_s1	Mm.PT.56a.29338718.g
RNF152	Hs00741870_g1	Mm.PT.58.29277956.g
RNF183	Hs00760214_s1	Mm.PT.58.42013627
RNF182	Hs00381370_m1	Mm.PT.58.30902864.g
TRIM59	Hs00416416_m1	Mm.PT.58.30417218
TRIM13	Hs00362364_m1	Mm.PT.56a.9751937
BFAR	Hs00275423_m1	Mm.PT.56a.28494992
RNF180	Hs00400379_m1	Mm.PT.58.45835696
RNFT1	Hs00211612_m1	Mm.PT.58.6836835
RNF26	Hs00259249_s1	Mm.PT.58.5764306.g
CGRRF1	Hs00198091_m1	Mm.PT.58.5664724
MUL1	Hs00226069_m1	Mm.PT.58.11670267
RNF217	Hs00332105_m1	Mm.PT.56a.33323466
